# The decline of malaria in Finland – the impact of the vector and social variables

**DOI:** 10.1186/1475-2875-8-94

**Published:** 2009-05-07

**Authors:** Lena Hulden, Larry Hulden

**Affiliations:** 1 Department of Forest Ecology, PO Box 26, FIN-00014 Helsinki University, Helsinki, Finland; 2 Finnish Museum of Natural History, PO Box 17, FIN-00014 Helsinki University, Helsinki, Finland

## Abstract

**Background:**

Malaria was prevalent in Finland in the 18th century. It declined slowly without deliberate counter-measures and the last indigenous case was reported in 1954. In the present analysis of indigenous malaria in Finland, an effort was made to construct a data set on annual malaria cases of maximum temporal length to be able to evaluate the significance of different factors assumed to affect malaria trends.

**Methods:**

To analyse the long-term trend malaria statistics were collected from 1750–2008. During that time, malaria frequency decreased from about 20,000 – 50,000 per 1,000,000 people to less than 1 per 1,000,000 people. To assess the cause of the decline, a correlation analysis was performed between malaria frequency per million people and temperature data, animal husbandry, consolidation of land by redistribution and household size.

**Results:**

*Anopheles messeae *and *Anopheles beklemishevi *exist only as larvae in June and most of July. The females seek an overwintering place in August. Those that overwinter together with humans may act as vectors. They have to stay in their overwintering place from September to May because of the cold climate. The temperatures between June and July determine the number of malaria cases during the following transmission season. This did not, however, have an impact on the long-term trend of malaria. The change in animal husbandry and reclamation of wetlands may also be excluded as a possible cause for the decline of malaria. The long-term social changes, such as land consolidation and decreasing household size, showed a strong correlation with the decline of *Plasmodium*.

**Conclusion:**

The indigenous malaria in Finland faded out evenly in the whole country during 200 years with limited or no counter-measures or medication. It appears that malaria in Finland was basically a social disease and that malaria trends were strongly linked to changes in human behaviour. Decreasing household size caused fewer interactions between families and accordingly decreasing recolonization possibilities for *Plasmodium*. The permanent drop of the household size was the precondition for a permanent eradication of malaria.

## Background

Vivax malaria was a common endemic disease in Finland in the 18^th ^and 19^th ^century and prevalent in the whole country. The situation was the worst in the south-western part with the archipelago [1,2]. The illness of the population was a major problem and much effort was made to study the disease [3-5]. According to contemporaneous reports by the district physicians, the mortality during malaria epidemics usually varied between 0.85 and 3% [1,5]. Malaria cases culminated in the spring and, in years with epidemics, the ploughing and sowing often remained undone in the villages [5]. The Rev. Eric Lencqvist was the vicar on the malarious island of Taivassalo (1750–1752). He explained that the whole household was sick on many farms and that several villages completely lacked healthy manpower [6]. Most authors naturally blamed the disease on the humid climate. The physician Johan Haartman, who visited the parishes in the archipelago, recommended that farmers should build their houses on windy cliffs and cut down all bushes and trees near them [7]. The advice was good, because that would have made the house less attractive for mosquitoes.

Malaria declined slowly in Finland without any deliberate counter-measures. The zoologist Johan Axel Palmén introduced in 1900 the international knowledge about the *Anopheles *species as vectors in Finland. He advocated for mosquito control, but he was unfamiliar with the ecology of the *Anopheles *species in Finland [8]. No effective control measures were, therefore, introduced. By then malaria had already declined to a quite low level and the last epidemic in Helsinki occurred in 1902 [9]. During the 1930's malaria was close to extinction.

The reasons for the decline have only been analysed superficially [10]. Swedish researchers usually refer to medication, improved hygiene and possibly the reclamation of wetlands as factors contributing to the decline of malaria in Sweden [11]. A recent review adds climate to the list of factors [12]. Previous research has mainly focused on the last decades of the decline of malaria, because of the lack of long-term series. The disease has therefore been related with environmental or social conditions that prevailed after the changes of the traditional agricultural society. As a consequence the apparent conditions during the last phase of malaria may point to misleading factors. An indication of this is the diversity of explanations for the decline that has been presented for various countries in Europe [13]. The short data series has also made it impossible to separate factors that had an impact on annual variations from those that influenced the long-term trend. In the present analysis of indigenous malaria in Finland, a data set on annual malaria cases of maximum temporal length was constructed for evaluating the significance of different factors assumed to affect malaria trends.

## Methods

### Statistical methods

The long term declining trend of malaria in Finland was compared with all variables which have been used as possible explanations for disappearance of malaria in various parts of Europe. Statistical analysis was performed only for those variables which are known to have changed significantly over the complete time period and have a reasonable causative relation to malaria prevalence. Several variables which are known to have changed only during a shorter time have been excluded from statistical analysis and are explained in the text.

### Malaria statistics

Statistics on malaria in Finland was collated from several sources. Malaria deaths in 1750 – 1850 were collected from the parish registers as described in Huldén *et al *[1]. Death cases for the year 1749 were manually collated from micro cards in the Library of Statistics in the Institute of Statistics, Finland. The relevance of this data set was statistically evaluated in Huldén *et al *[1]. Since malaria had almost disappeared from Finland before World War II, the Karelian area ceded to Soviet Union in 1944 is included in the study.

Because the digitalized data of the parish registers extend only partially after 1850, the annual reports by district physicians collated by Sievers in 1891 [5] were used to reconstruct the annual number of malaria cases between 1850 and 1885. These assessments were linked to the previous number of death cases by multiplying the death cases with a factor of 50, assuming a mean mortality of 2% during the time when practically no medication were in use. This is in accordance with the annual reports of the district physicians [5]. The reconstruction has a slightly lower annual variability than the actual malaria epidemics, but is expected to correctly reflect the overall trend.

The official statistics on malaria improved in the 1880s and were used for the years 1884 to 1952 [14]. The last known indigenous cases occurred during the years 1953 to 1954 [15]. Imported malaria commenced in the 1960s in Finland. Data for the years 1968 to 1970 and for the years 1971 to 2000 represent only imported cases [15,16]. The most recent data for 2001 to 2008 are available online [17].

**Figure 1:**
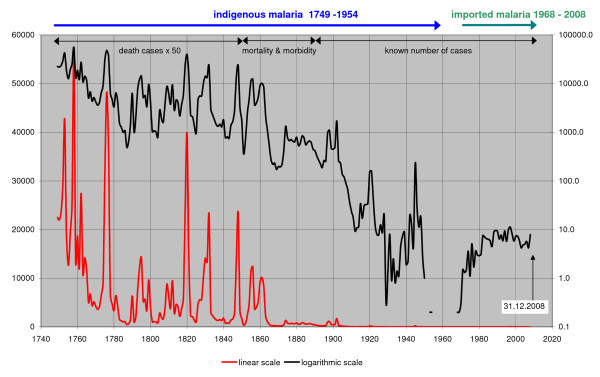
**Annual malaria frequency trends in Finland in 1749–2008, expressed as cases per 1,000,000 population on a linear and on a logarithmic scale**. Values for death cases in 1749–1849 are corrected for incompletely available parish data.

Annual cases were transformed into an index of number of cases per million people to obtain the true trends in malaria frequency. It is presented in Figure 1. All correlation analysis of malaria with other factors was performed for three time periods, 1750–1830, 1830–1890 and 1890–1960. The end year of the first period is adjusted because temperature data for Helsinki starts only in 1829.

**Figure 2:**
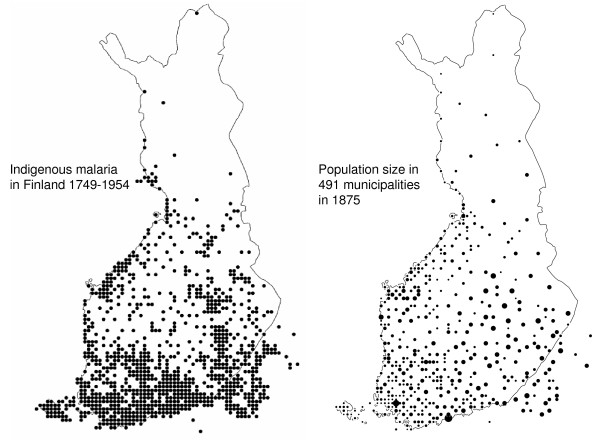
**Left map**. Distribution of cumulative indigenous malaria cases Finland in 1749–1954. Dots represent 10 × 10 km^2 ^units. Right map. The size of population in the municipalities in 1875 is presented according to the relative size of dots. The distribution of malaria comprised nearly all the inhabited parts of Finland.

**Figure 3:**
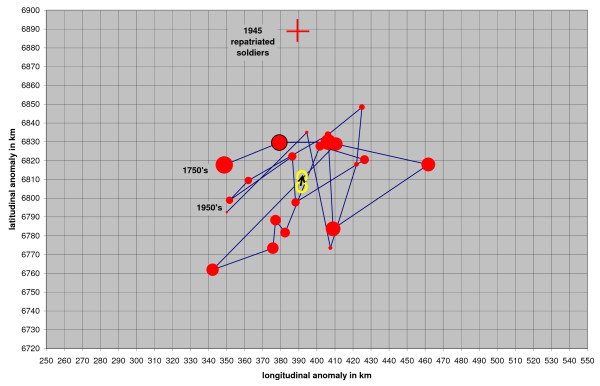
**Decadal trend of equilibrium points of malaria localities in 1750 – 1959**. The linear trend line from the end points in 1750's and 1950's are indicated with yellow rings. The length of the black vector is only 4,9 km. The decreasing size of red dots symbolizes decreasing malaria frequency.

A cumulative map for all known indigenous malaria cases in Finland in 1749 – 1954 is presented according to 10 × 10 km^2 ^units (Figure 2). For comparison a map of the population density in 1875 in Finland is added. Malaria occurred over the whole country with the highest density in the south-west. The decadal distributional trends of malaria are presented in 24 maps [see Additional file 1]. The last fifteen years are presented in four and five year parts and the year 1945 separately. The maps include a red cross representing the equilibrium point of all dots on each map. The decadal trends of the coordinates of the crosses are shown in Figure 3.

### Other statistics

Historical trends of various factors were compiled from several sources:

1. Official demographic statistics have been collected since 1749 [15,18].

2. Changes in animal husbandry were estimated by the number of cows on each farm. Cattle were a part of the annual taxation and the statistics are, therefore, available [18].

3. Most of the land in Finland was owned by independent farmers. The great redistribution of land holdings started in the 1760s. It was later completed with further land consolidations. The data was calculated as the annual number of partitions made by the official land surveyors [19,20].

4. Statistics on household size in 1749 – 1860 was calculated from published statistics on the number of farms and rural population size [18]. A small linear adjustment for urban population was performed based on known values for 1749 and 1800. The urban population constituted only 4–6% of the total population in Finland and had little impact on the whole statistics. Statistics on household size in 1860 – 1960 has been published [21]. Statistics on the number of persons per room is published for the time period of 1860–1960 [21]. The time period of 1750–1860 was interpolated backwards from 1860 assuming a slow change in the average number of rooms per house which was on average only 2.09 in 1860.

5. Statistics on temperature trends for Uppsala and Stockholm (Sweden) was used for 1749 – 1959 and 1756 – 1959 respectively [22]. Temperature data for Helsinki (Finland) is available only from 1829 [23].

6. Statistics on reclamation of wetlands is available for 1920 – 1990 [24].

7. Lowering of water level in lakes needed permission and was performed in 1750's – 1890's [25].

The distribution of the *Anopheles *species in Finland is reasonably well known [26].

The determination of *A. messeae *was verified by molecular sequences by Gunilla Ståhls-Mäkelä, Finnish Museum of Natural History.

## Results and discussion

### Decline of malaria

The decline of malaria commenced in the latter half of 18^th ^century. The total distributional shift of the linear regression trend during 200 years is only 4.72 km northwards and 1.21 km eastwards, i.e. about half percent of the total extension of malaria in Finland (Figure 3). Unweighted least squares linear regression gives a 90% confidence interval of +/- 19.3 km during 200 years. As a consequence the statistical drift of the equilibrium point does not differ from zero. In the same time, malaria frequency decreased from about 20,000 – 50,000 per 1,000,000 people to less than 1 per 1,000,000 population. In other words, malaria faded out evenly over the whole country.

### The impact of the vector on malaria in Finland

Malaria in Finland was an "indoor" disease and the main transmission season lasted from December to May [1]. The vectors were *Anopheles messeae *and *Anopheles beklemishevi*. The same species have been identified as the main vectors in northern Russia [27,28]. The third Finnish species, *Anopheles claviger*, can be ignored as it is rare in the southernmost part of Finland [29]. The females of *Anopheles messeae *and *A. beklemishevi *oviposit in May – June and the larvae develop during June and most of July. The hatching of imago peaks in the first half of August [1]. Adult anophelines are absent from a survey of mosquitoes made in early July [30]. In the sampling outdoors made by the authors in 2008, the first adult *A. messeae *were collected at the end of July. In the mid- and south taiga region of Russia, *A. messeae *has two, less often three, generations per year [28]. In Finland, it usually only has one generation per year. During very warm summers in southern Finland *Anopheles messeae *may mate and lay eggs also in July.

Decadal distribution of malaria cases oscillates stochastically along a maritime – continental gradient (from southwest to northeast) with a statistically negligible northeast drift. The oscillation is explained by regional variation of weather conditions affecting regional variation of mosquito frequency. *Plasmodium vivax *survives in humans as hypnozoites during unfavourable times and that is why it has a relatively stable overall distribution. Considering the combination of spatially stable distributional trend but temporally declining trend of malaria the vectors are excluded as an explanatory factor of the decline. Any long-term changes of environmental factors related to the mosquitoes are most likely causing a geographical drift in the distribution of the vector species, but this drift is so small that it is not visible in the long-term malaria distribution.

**Figure 4:**
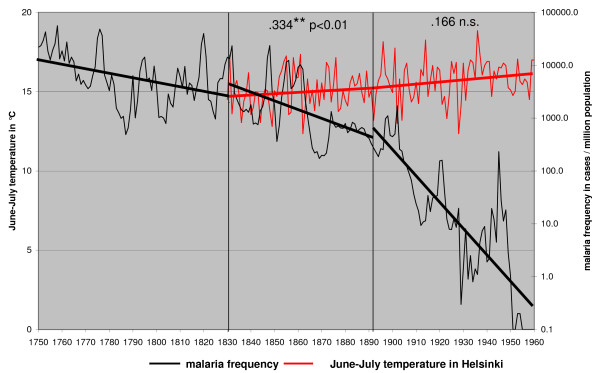
**June – July temperature in 1829 – 1960 from Helsinki (Finland) and the annual malaria frequency trends in Finland in 1750 – 1960**.

**Figure 5:**
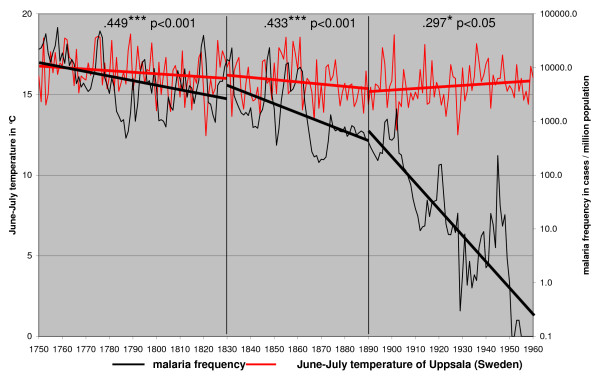
**June – July temperature in 1749 – 1960 from Uppsala (Sweden) and the annual malaria frequency trends in Finland in 1750 – 1960**.

**Figure 6:**
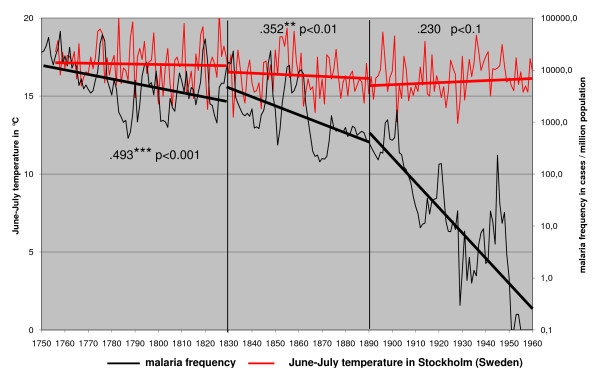
**June – July temperature in 1756 – 1960 from Stockholm (Sweden) and the annual malaria frequency trends in Finland in 1750 – 1960**.

The only large scale factor that could have affected long-term vector frequency trends without affecting the distribution would be changes in the mean temperatures. Annual frequency of malaria was compared with June–July temperatures from Helsinki, Finland (Figure 4), Uppsala, Sweden (Figure 5) and Stockholm, Sweden (Figure 6). Only June–July temperatures have been shown to have an impact on malaria frequency [1]. The annual mean temperature is in this case meaningless.

The correlation of malaria frequency and temperature is highest in the first correlation period (1750–1830 as defined in the methods) and step wise decreasing in the second (1830–1890) and the third correlation period (1890–1960). This correlation pattern coincides with the changing steepness of the decline of malaria. High peaks in malaria followed high temperatures in June–July [1]. Still malaria trend is declining although summer temperatures are stable or increasing especially in the third correlation period (1890–1960). Both highs and lows in malaria frequency are declining independently of temperature trends.

Summer temperatures regulate the development of the larvae and determine the number of overwintering *Anopheles *females inside the houses [1,31]. A second generation of *A. messeae *probably occurred during very warm summers which explain occasional high peaks in spring malaria. Only in exceptional conditions, could an epidemic be explained independently of the temperature. Such an occasion was the epidemic among Finnish soldiers during World War II [32].

Finland had a very sparse population in 1750, 1.2 people/km^2^, which increased to about 5.2 people/km^2 ^in 1870 when changes in rural practises commenced [18]. The official proportion of urban population increased from 4.3% in 1750 to 12.8% in 1900 [18].

The human impact on the water quality in the Finnish coastal waters was practically non existent before the middle of the 20^th ^century [33,34]. Because of the uplift of the land, the shores of the sea are gradually moving. The ecology of the shallow water and the shores therefore remains stable. Artificial fertilizers were sparingly used only from the 1870s onwards [35].

The lakes in the rural areas showed no distinct environmental disturbances and diatom and Chironomidae (Diptera) species composition in lake sediments remained virtually unchanged until the early parts of the 20^th ^century [36]. As a consequence we may also conclude that there have been no significant changes in predators either. The impact of paper and sulphite mills remained low in bigger lakes until the 1920s [37]. Even an urban lake (in the town of Jyväskylä) preserved the Benthic Quality Index in a pre-industrial state until 1930s [38]. The conclusion is that the vector species populations have not or only marginally been affected by environmental disturbances during the decline of malaria in Finland.

### Changes in animal husbandry, lowering of lakes and the reclamation of wetlands

**Figure 7:**
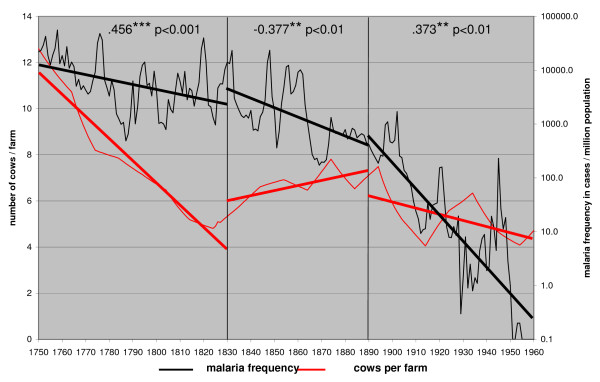
**The number of cows per farm and the annual malaria frequency trends in Finland in 1750 – 1960**.

The eradication of malaria in Denmark has been explained by a change in the feeding behaviour of the anopheline vectors, which was due to the modernization in animal husbandry between 1860 and 1880 [39,40]. If cattle and pigs were kept inside modern cowsheds and pigsties the whole year around, it is assumed that the anophelines changed from being antropophilic to zoophilic [39,40]. Finland was also a rural country [18] and there were little changes in agricultural practices during 1750s to 1870s [41]. The trends in the number of cows per farm was used as a proxy for a possible changing impact of cows on malaria frequency, There is a high positive correlation with malaria frequency in 1750–1830 (Figure 7). During this time manure was essential as fertilizer in areas with permanent fields and indirectly also determined the size of the farm and the household [42]. The correlation coefficients for the three time periods, however, are highly contradictory, suggesting that the cows are irrelevant for predicting long-term trends in malaria frequency.

The modernization in Finnish agriculture started in 1870's [43]. The production became concentrated on animal husbandry and corn was largely imported from Russia [41,43]. By then, rural malaria frequency had already declined to about 1% of the levels in the middle of the 18^th ^century. Compared with Denmark, the changes in animal husbandry were different. The cows were still kept outside during the summer and the main change was made in breeding, feeding during winter and in the modern constructions of cowsheds [42]. The practise of keeping cattle outside in pasture far from the farm during summer remained important still at the beginning of the 20^th ^century [35]. A change from antropophily to zoophily would have been impossible in the Finnish cottages because the overwintering anophelines had no other choice than to suck blood from human hosts. Still malaria declined at an accelerating rate.

Lowering of water level of lakes coincides largely with the main decline of malaria in the 18^th ^and 19^th ^century. This factor, however, was not affecting coastal or archipelago regions where the highest frequency of malaria is documented. Compared with the number of lakes in Finland, the practise did not have a noticeable impact on the Finnish landscape. There are 180,000 lakes in Finland and only 1,500 smaller lakes were lowered [25]. A comparison of historical maps with modern maps of some malarious parishes showed no noticeable temporal or spatial changes in the extent of the lakes which could have had a major impact on mosquito populations or correlated with the decline of malaria. Thus this factor can also be ruled out because malaria declined evenly over the whole of country.

**Figure 8:**
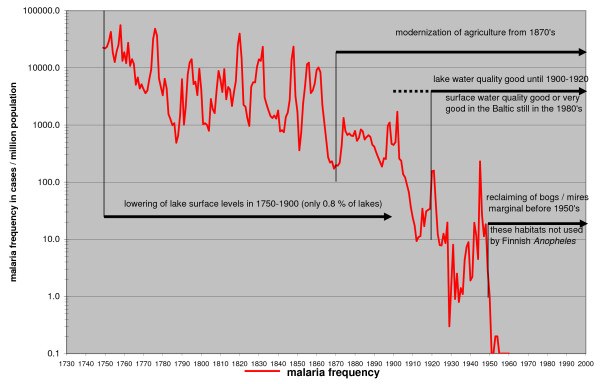
**Timing of factors considered irrelevant for malaria decline in Finland discussed in the text**.

Originally about a third of Finland consisted of wetlands [44]. The reclaiming of wetlands for agriculture started at the end of the 19^th ^century. It still remained very limited before 1950's and only about 3 – 4% of all wetland area was dried. In 1950 – 1980, about 65% of the wetlands have been affected. Wetlands can be ruled out as a contributing factor because of wrong timing in relation to the decline of malaria (Figure 8).

### Social factors as an explanation for the decline of malaria in Finland

**Figure 9:**
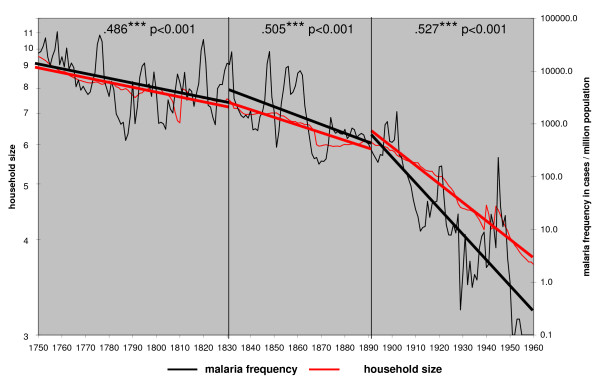
**Household size and the annual malaria frequency trends in Finland in 1750 – 1960**.

**Figure 10:**
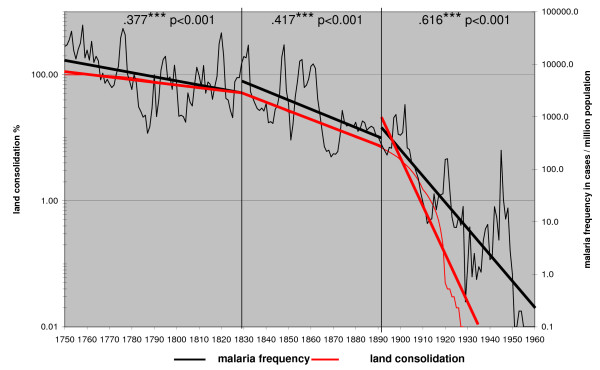
**The land consolidation process expressed as % of unconsolidated land in relation to consolidated land in 1920 (when the process was practically finished) and the malaria frequency trends in Finland in 1750 – 1960**.

The view on malaria as a social disease is not new. Angelo Celli thought that economic and social reforms combined with the widest possible use of quinine were the best way to fight malaria in Italy [13]. De Zulueta has stated that the eradication of malaria from Europe and North America was much more due to changed social conditions than the use of insecticides [45]. The Finnish data offer an opportunity to test social factors with the decline of malaria. The declining trend shows that the factors with an impact on the long-term series were variables that affected the disease gradually and evenly throughout the whole country. The average household size and consolidation of land by redistribution show both a high correlation with the decline of malaria (Figure 9 and 10). Neither the vector nor the temperature had an impact on the long-term trend. The use of quinine was limited in Finland before 1850 [1]. The drug started to be used more effectively in 1857–1865. Then, during an epidemic, the district physicians could require that it would be distributed free among the poor. Paradoxically, the independent farmers did not want to spend money on quinine and their households continued to suffer from the disease [46]. To make cheap quinine available for everybody, as was done in Italy [47] or in the Netherlands [48], was never an issue in Finland.

The infected vector needed to be able to transmit the parasite to uninfected humans and malaria in Finland was only transmitted indoors during winter by nocturnal *Anopheles *species [1]. The sleeping habits of the human hosts were therefore crucial. The survival of *Plasmodium vivax *depended on uninfected people who slept in the same room together with a carrier of the parasite and a potential vector. In 1750, the average number of household members was 9.58; it decreased to 5.83 in 1900 and in 1950 to 4.03 [21]. The land consolidation process changed the distribution of farms in the Finnish landscape during the same time [19,20].

The great redistribution of land holdings started in the 1760s in Finland [19]. It broke the old medieval open field system and replaced it with independent farms. The houses were first built close to each other. The purpose of the land consolidation was to redistribute the land into larger units. This also made it easier to found new farms and crofts. It was largely finished in the 1920s and the process correlated closely with long term malaria trends in Finland (Figure 8). The farmers gradually started to move the buildings of the farm from the old traditional village to their own land. A change to separate farm units made the ownership of the land more explicit and had an impact on the family structure. The extended family household was gradually replaced by a nuclear family household [49]. The process of land consolidations had also social consequences for the village community. It created legal fights over land between the villagers and the patterns of interaction in the parishes changed. The traditional rural parish, where villages formed the social base, was replaced by a society in which family ties were formed by farmers on the ground of property [50-52].

The household size in Finland declined slowly from the end of the 18^th ^century onwards. During the later part of the 19^th ^century, the living standard among the landless people started to rise. They could more often afford their own cottage on rented land [53]. The trend of the declining household size is similarly strongly correlated with declining malaria frequency in Finland (Figure 9). Malaria finally disappeared when the household size approached four members and below. A smaller household naturally meant fewer people sleeping in the same room.

**Figure 11:**
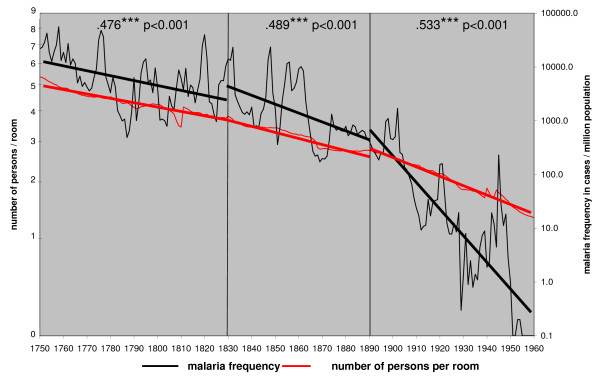
**The number of persons per room and the annual malaria frequency trends in Finland in 1750 – 1960**.

The correlation of malaria frequency and the number of persons per room is also very high for all three time periods (Figure 11). After 1850 the farmers could gradually afford houses with more than one bedroom. It became more common for the maids and the hired hands to sleep and eat separately from the family [53]. The possibility for the vectors to transmit malaria to or from the servants diminished.

The main result is that in Finland, where no mosquito control was used, the combined factors related to humans are relevant, but factors related to mosquitoes are irrelevant in affecting the long-term decline of malaria. Land consolidations and household size showed a high correlation with the long-term malaria trend (Figure 8 and 9). The process of land consolidation changed the integrated extended family community into a nuclear family community. New farms were founded and the household size declined. This sociological change irreversibly shifted the balance between the rates of extinction and new colonization for the *Plasmodium*. A habitat fragment (a patch) for *P. vivax *was comprised of all the humans sleeping in the same room together with the vector. The nuclear family and a smaller household size made that habitat smaller and more isolated. A colonization event for the *Plasmodium *was a situation when a new person arrived in a household. It could be a hired hand, a temporary guest or a child from a neighbour sleeping over. In the rural community of the 18^th ^century, visitors would have slept with the family. The development of the nuclear family in a small household changed the old patterns. The bedroom had become a private space. The possibility for the *Plasmodium *to colonize a new habitat diminished.

## Conclusion

The indigenous malaria in Finland faded out evenly in the whole country during 200 years with limited or no counter measures or medication. This represents one of the very few opportunities where natural malaria dynamics can be studied in detail. The mosquito population can in this case be ruled out as an explanatory factor in the extinction process of malaria. It appears that malaria in Finland basically was a sociological disease and that malaria trends were strongly linked to changes in the human household size and housing standard.

It must be emphasized that it is the size of the vector population that determined the large annual variations of malaria frequency. The Finnish data showed how the situation developed without mosquito control. The reduction of the vectors indoors would probably largely have decreased the economic impact of malaria epidemics on the society. In that case, the declining trend would have been faster and the housing standard of the human population would have improved faster.

## Competing interests

The authors declare that they have no competing interests.

## Authors' contributions

LEH drafted the manuscript and collected the historical data. LAH constructed the maps and performed correlation analyses. Both authors read and approved the final manuscript.

## Supplementary Material

Additional File 1 **Decadal distribution of malaria in Finland in 1750–1954.** A red cross represents the equilibrium point of all dots on each map. Click here for file
