# Supplementary figures and images for: The decline of malaria in Finland – the impact of the vector and social variables
Source: Malar J. 2009 May 7;8:94. doi: 10.1186/1475-2875-8-94 (PMC2684538; doi:10.1186/1475-2875-8-94)

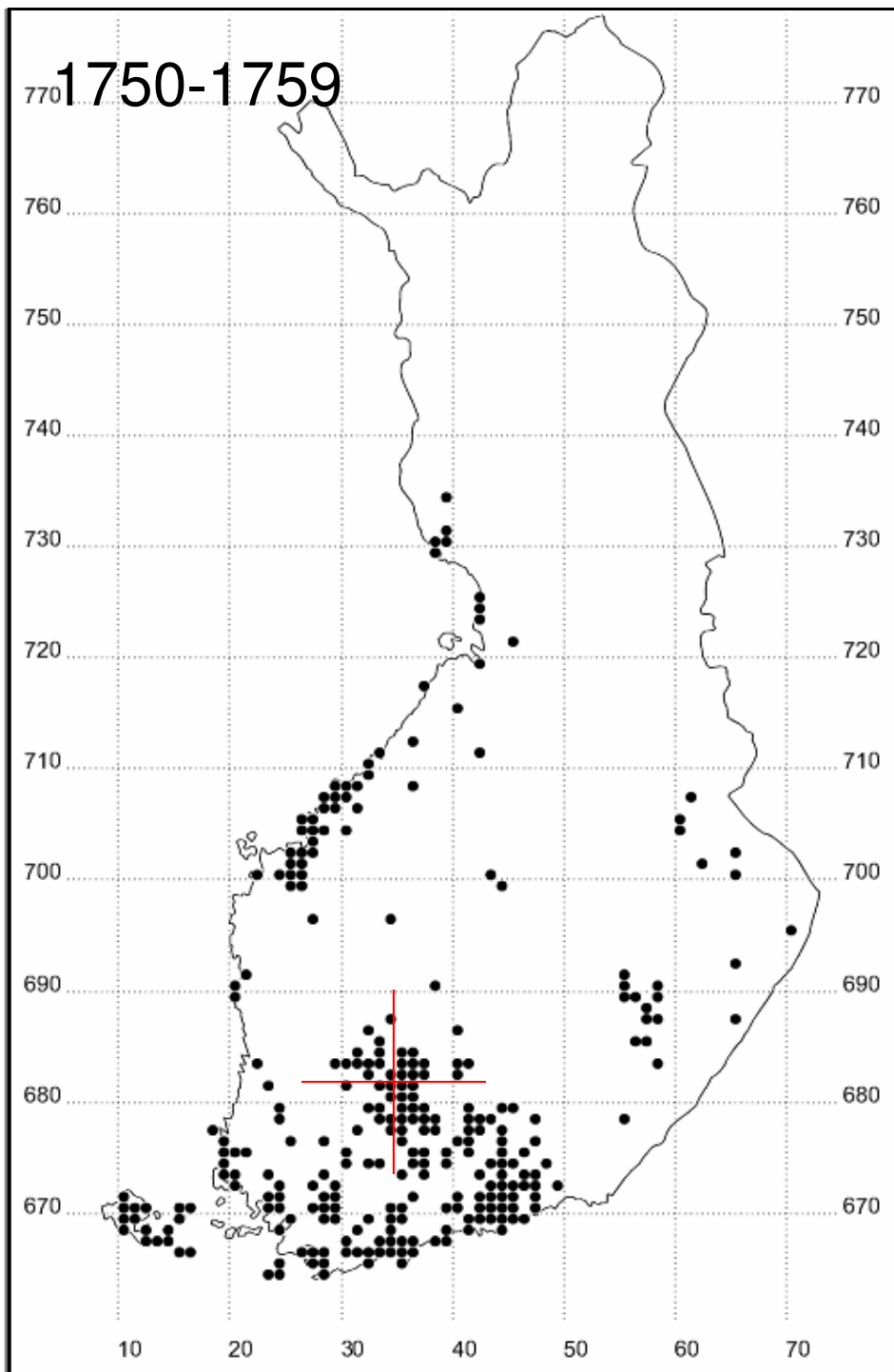

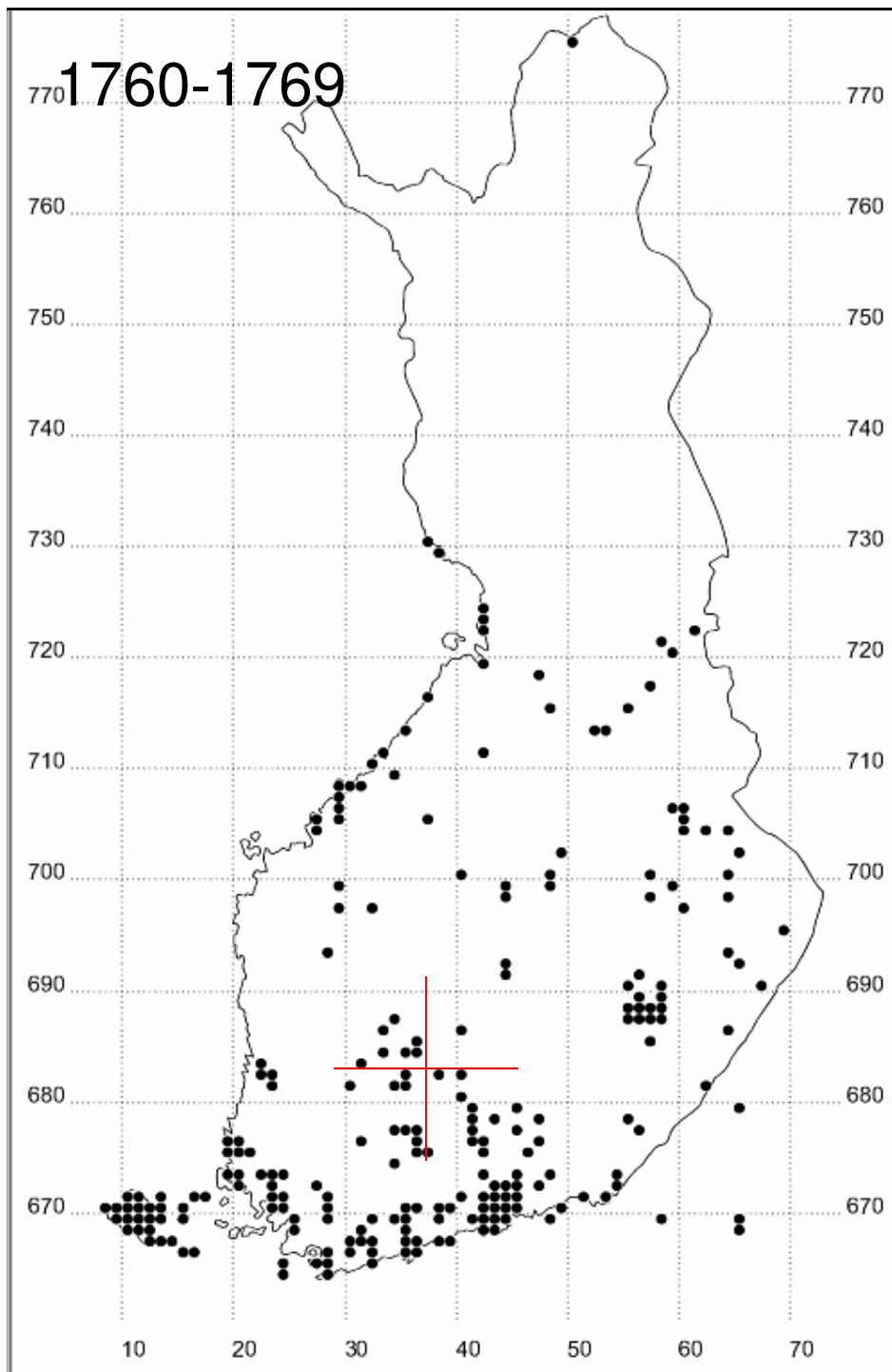

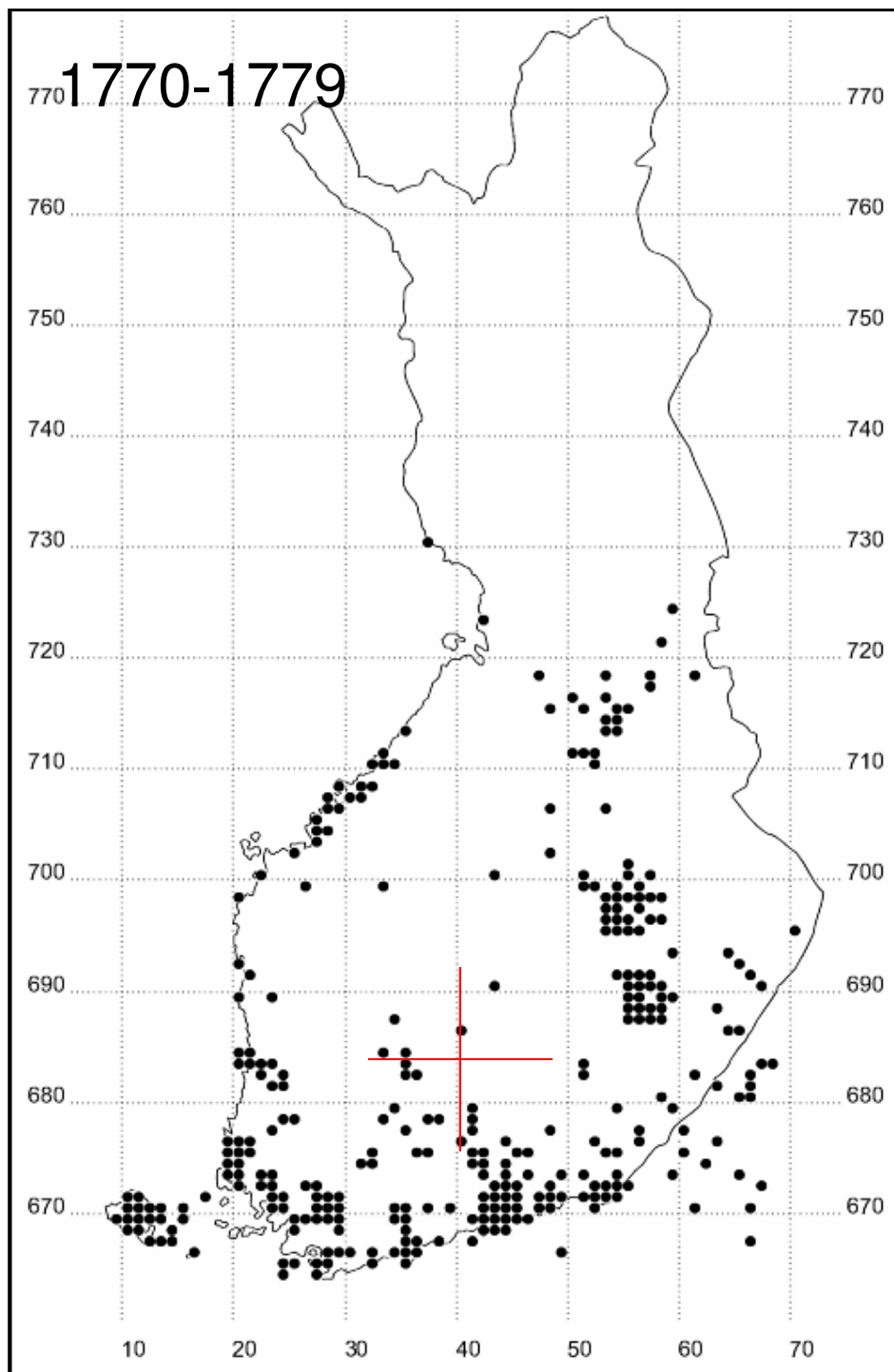

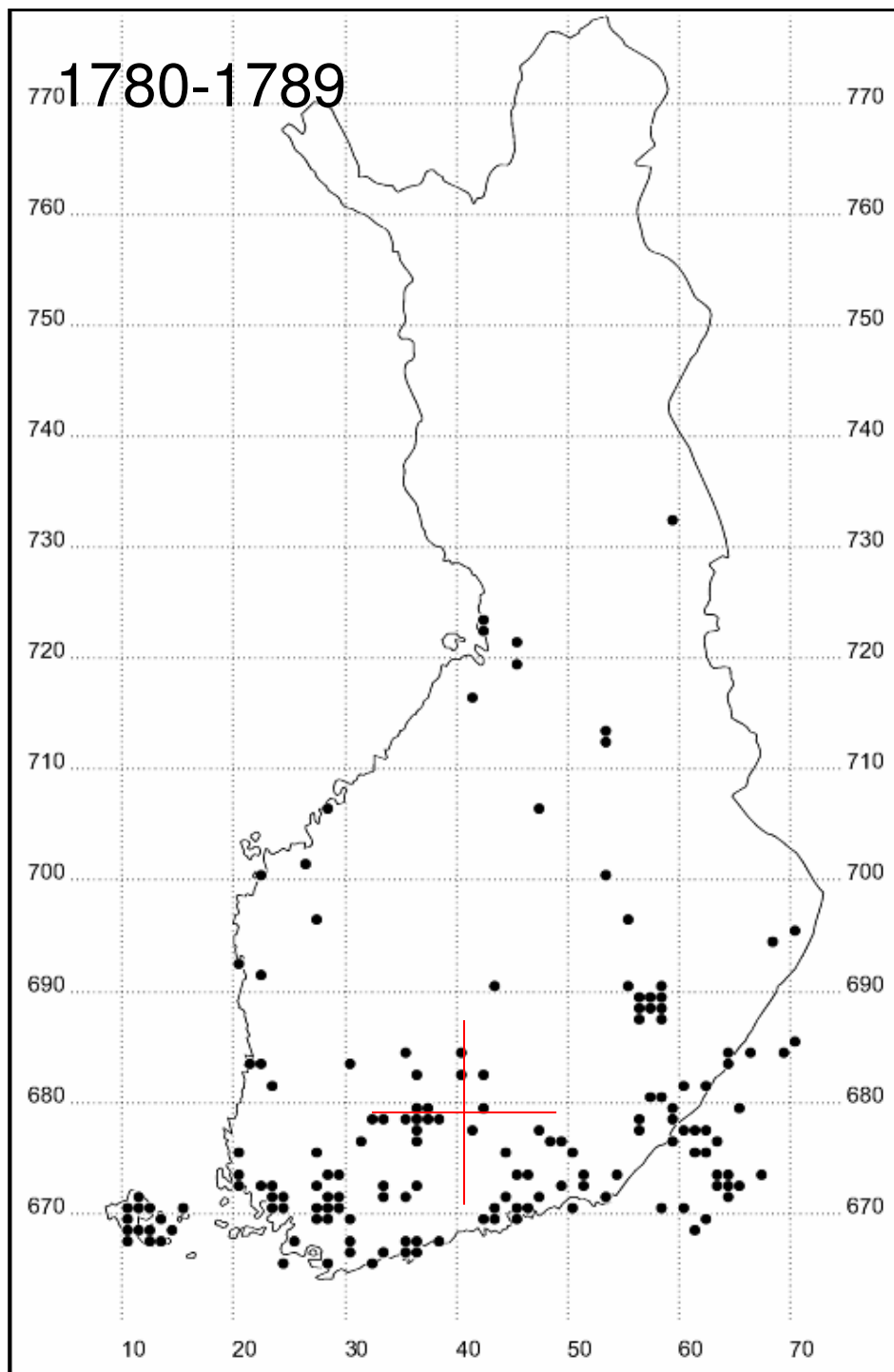

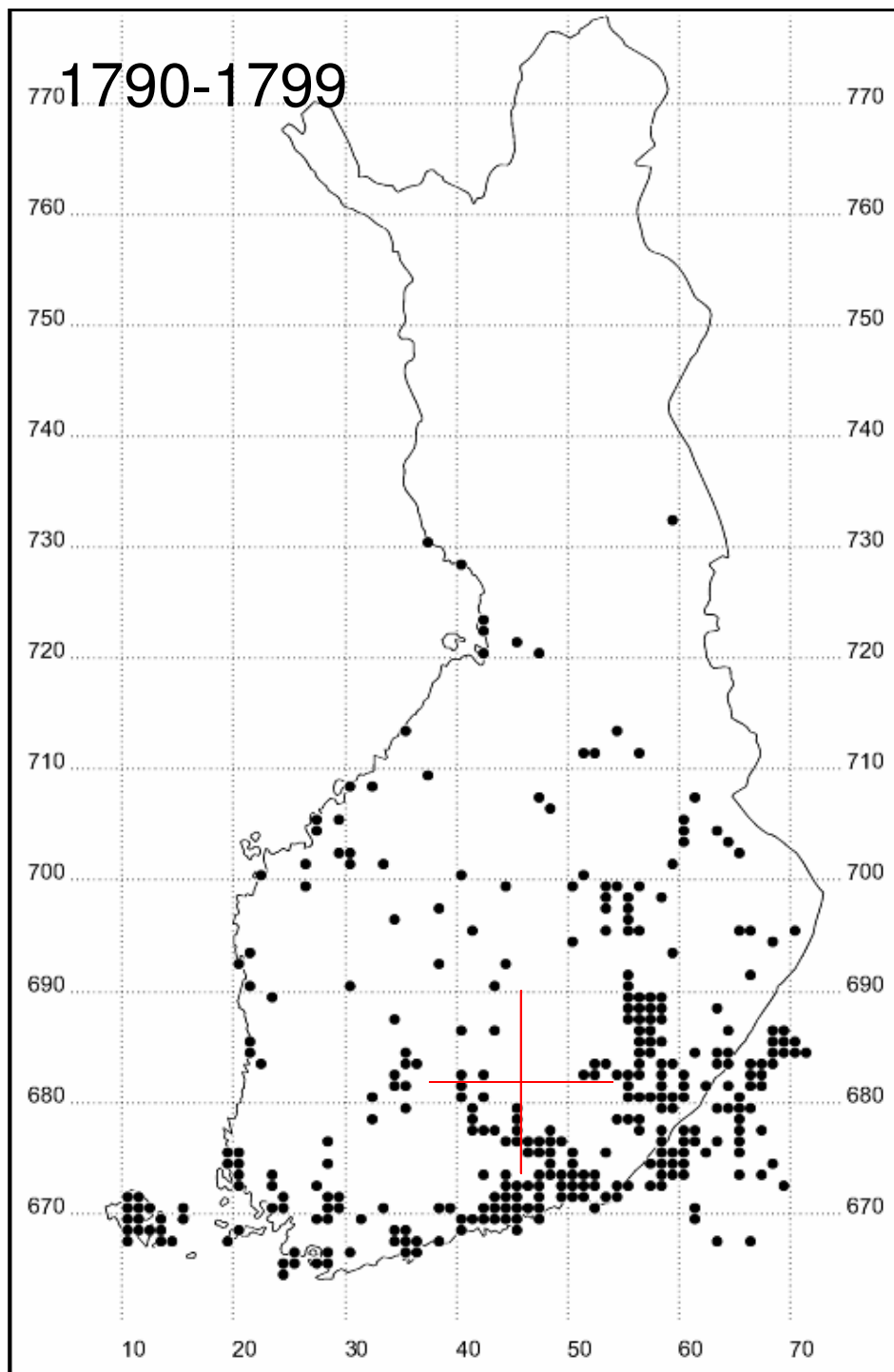

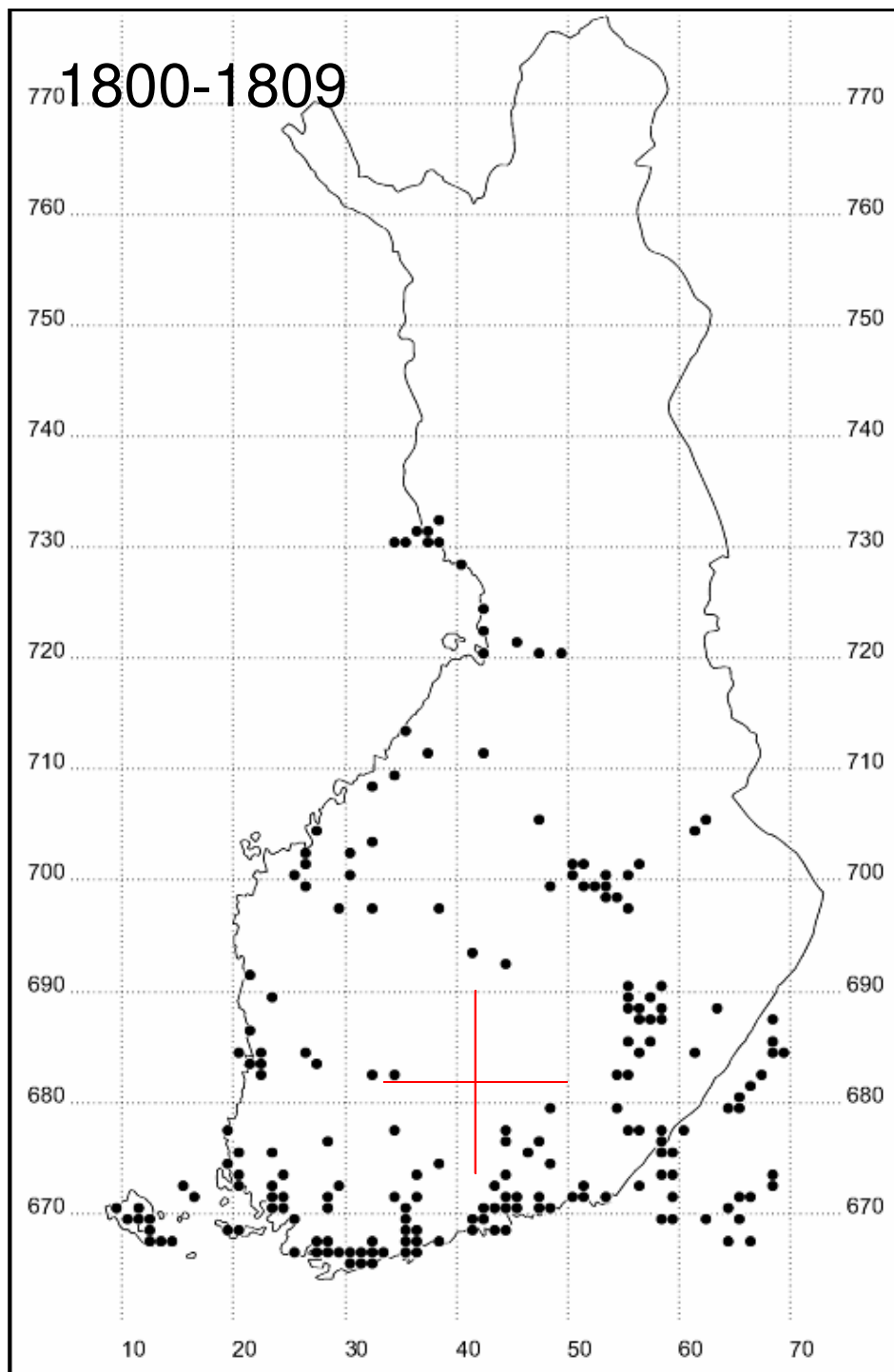

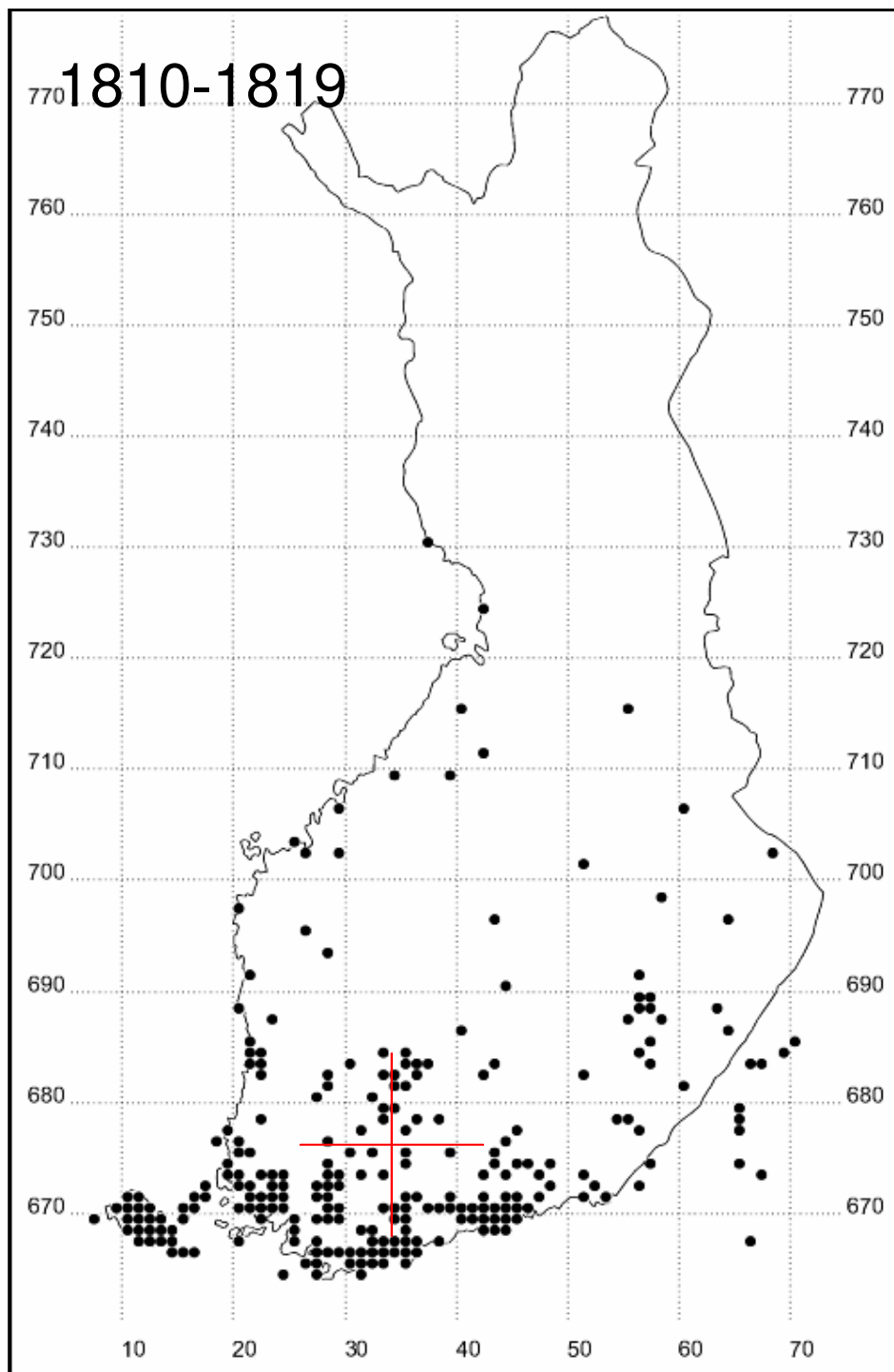

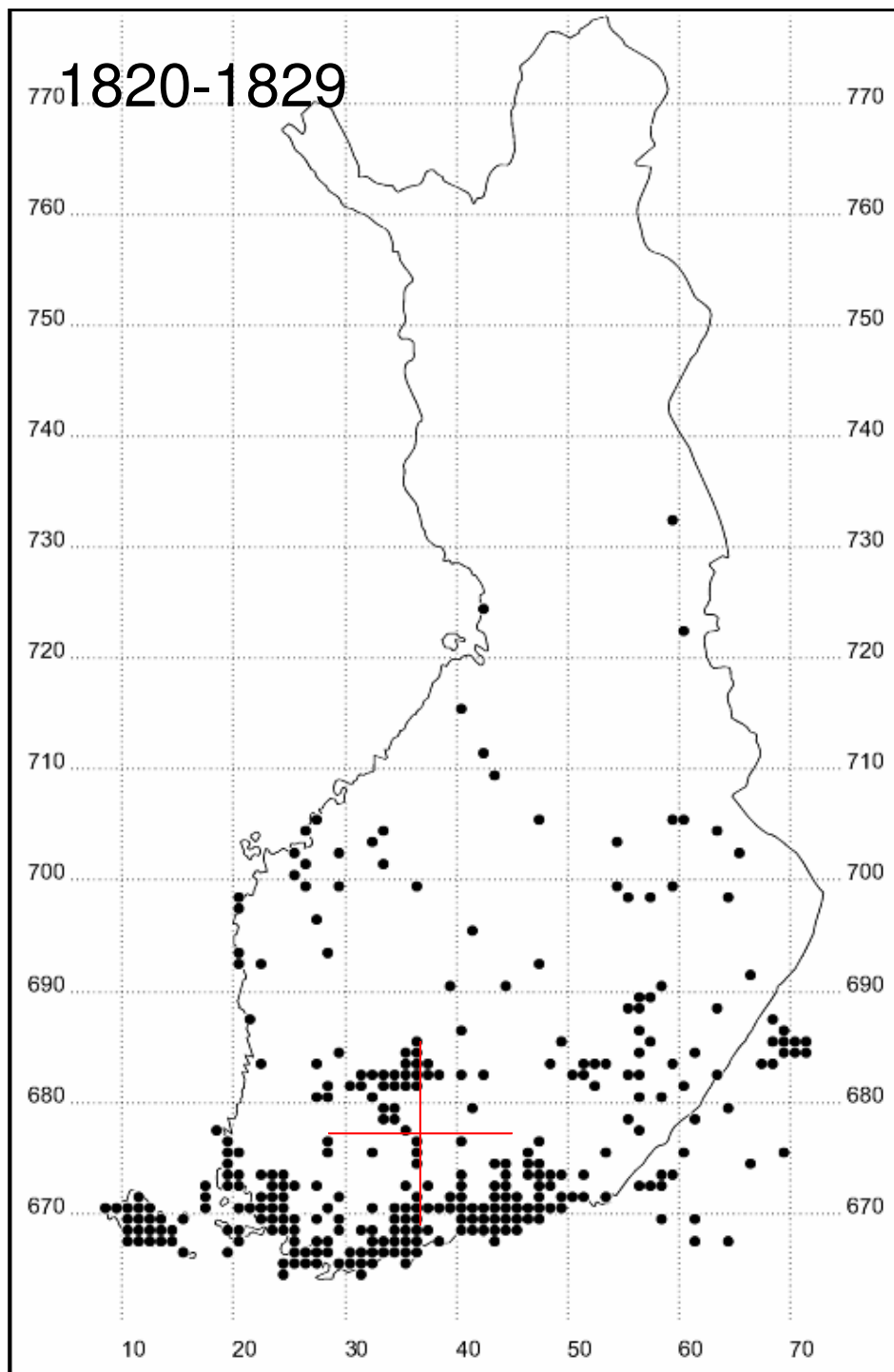

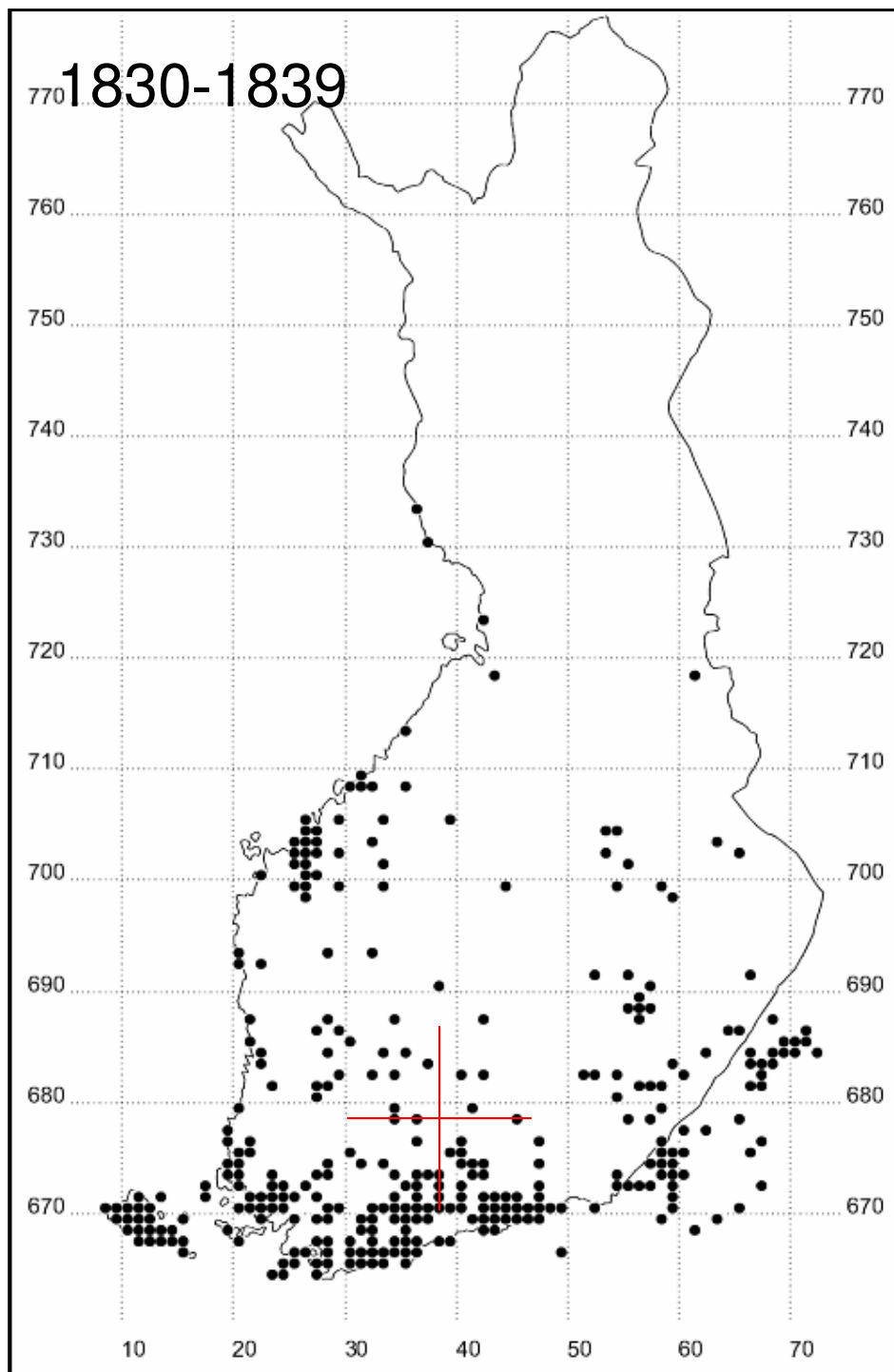

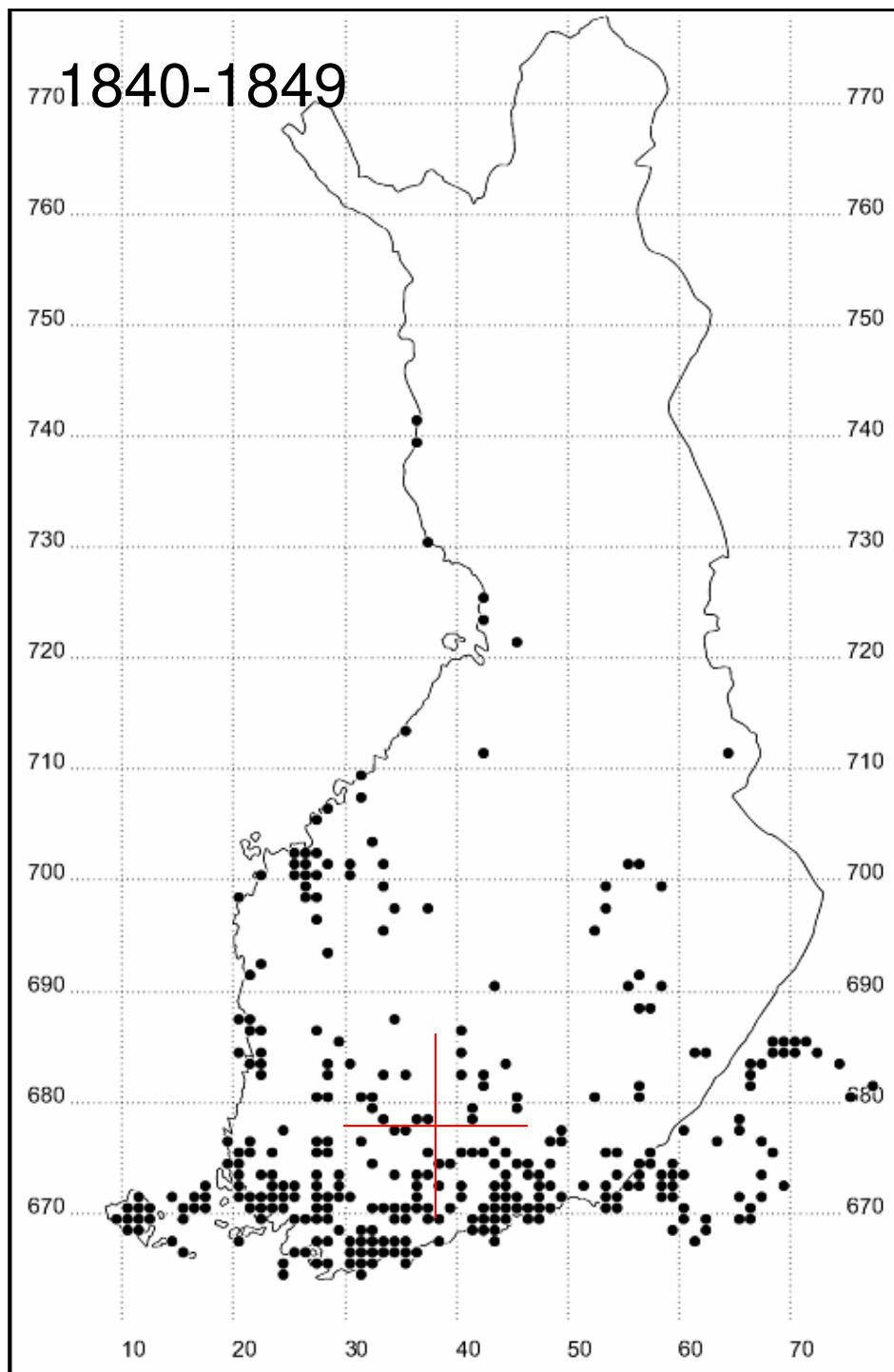

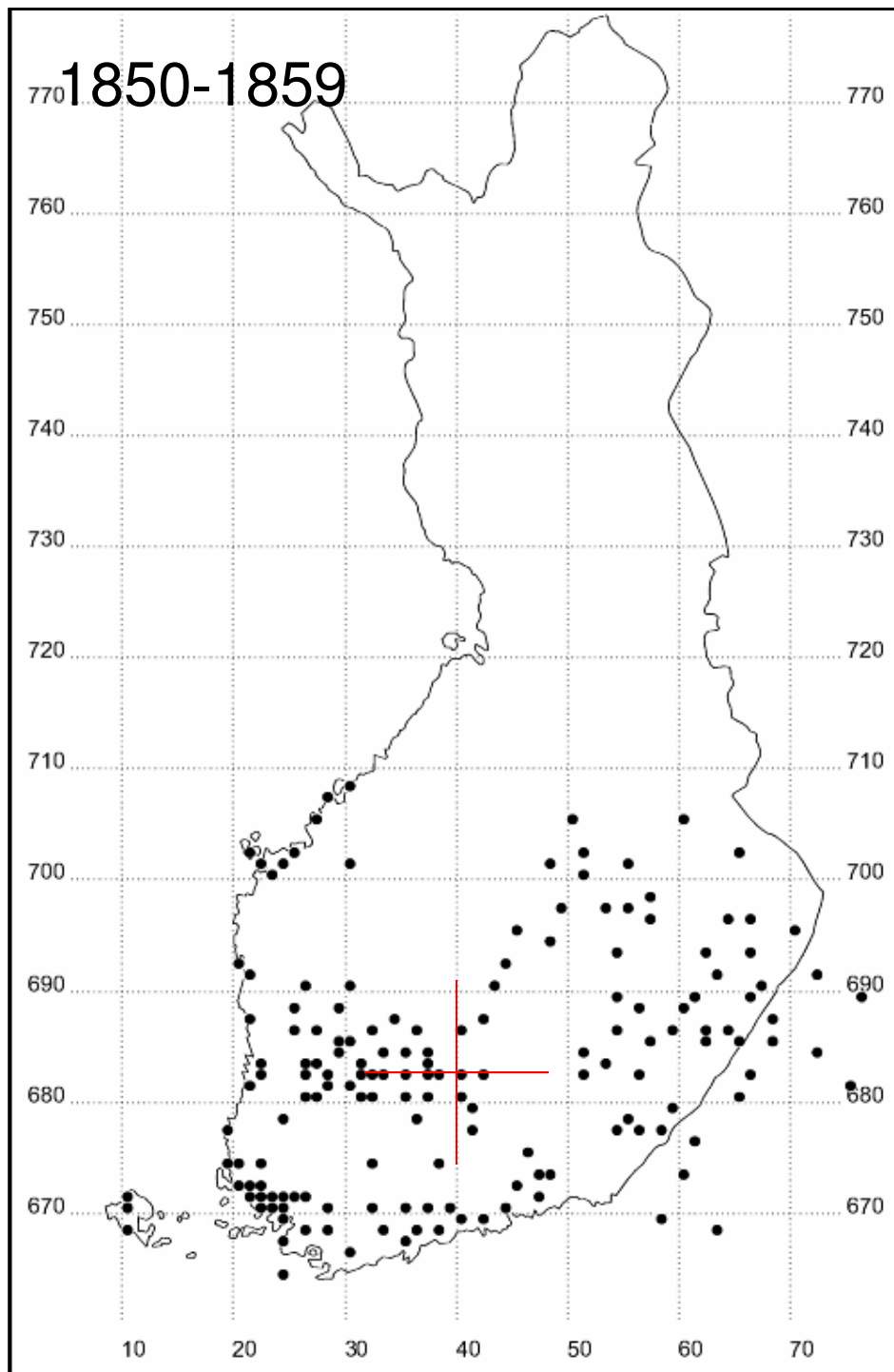

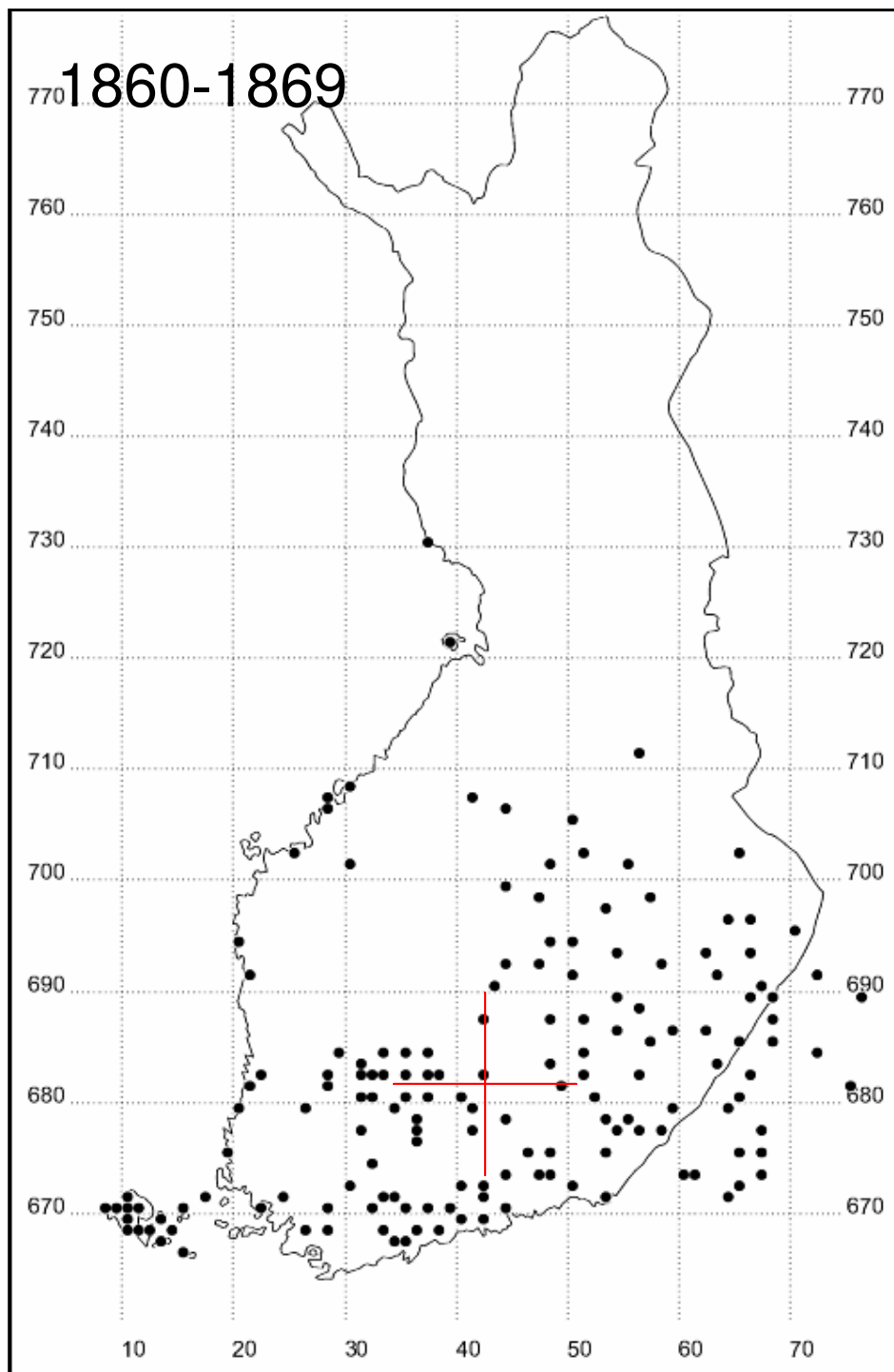

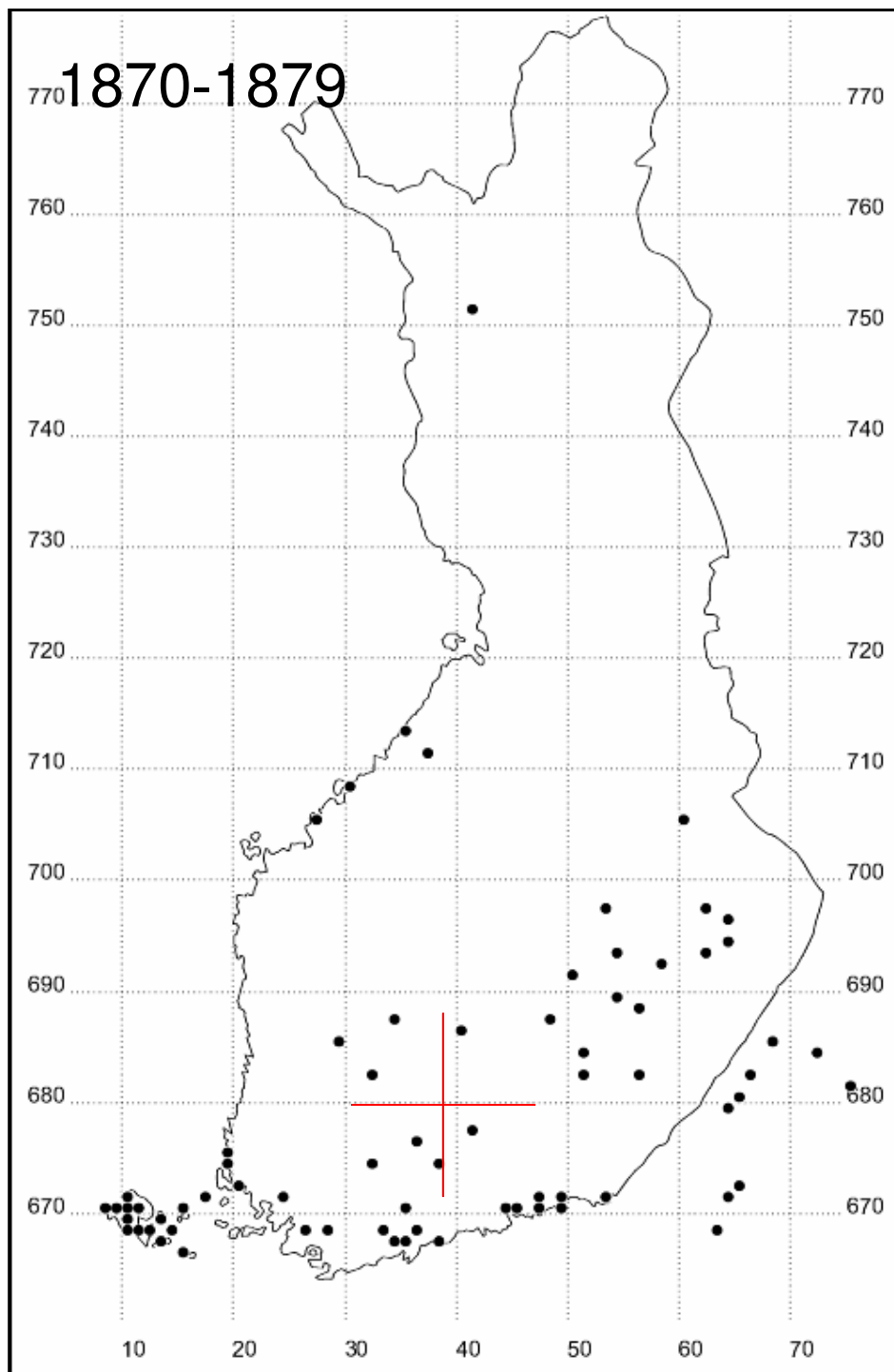

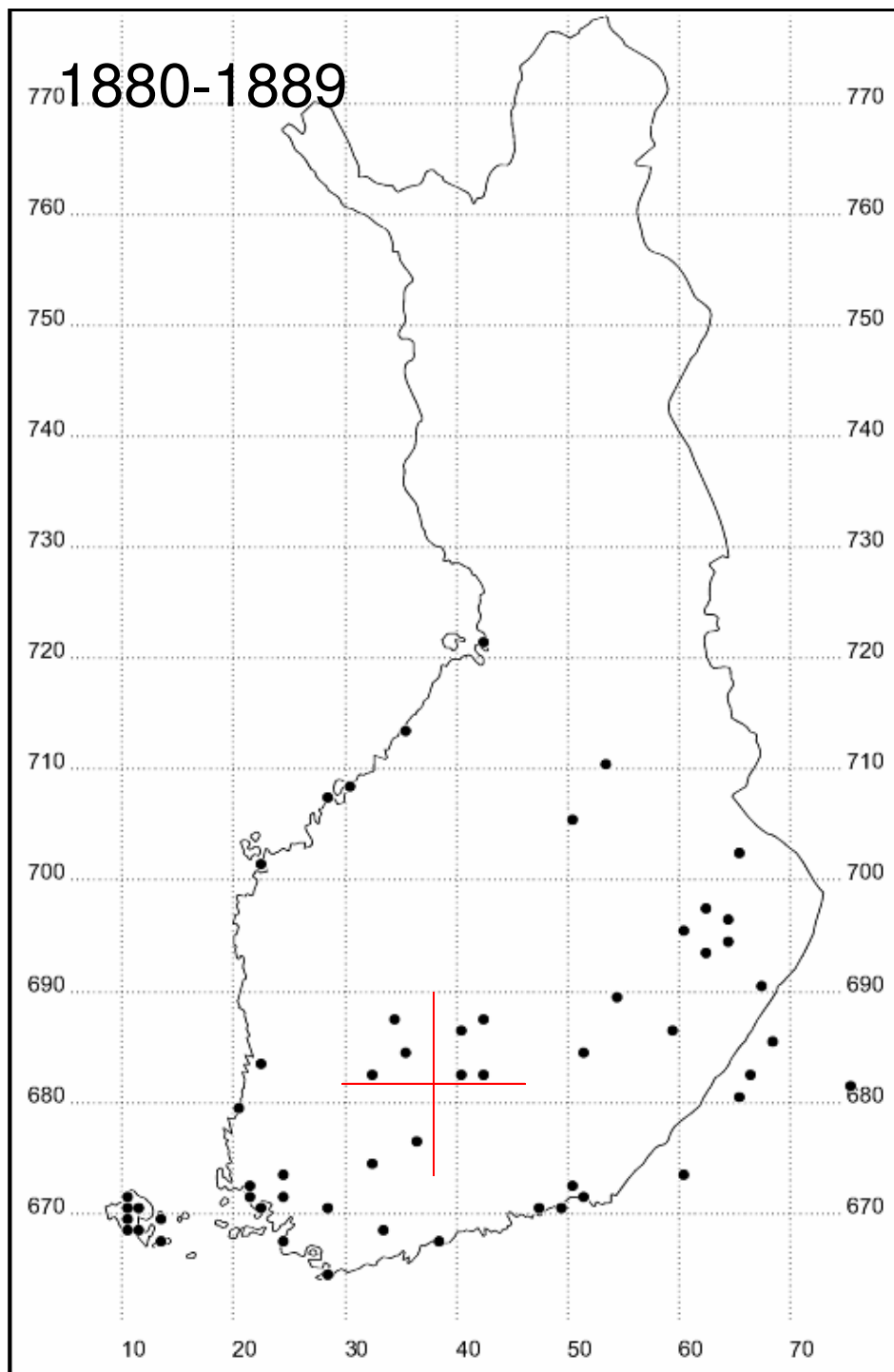

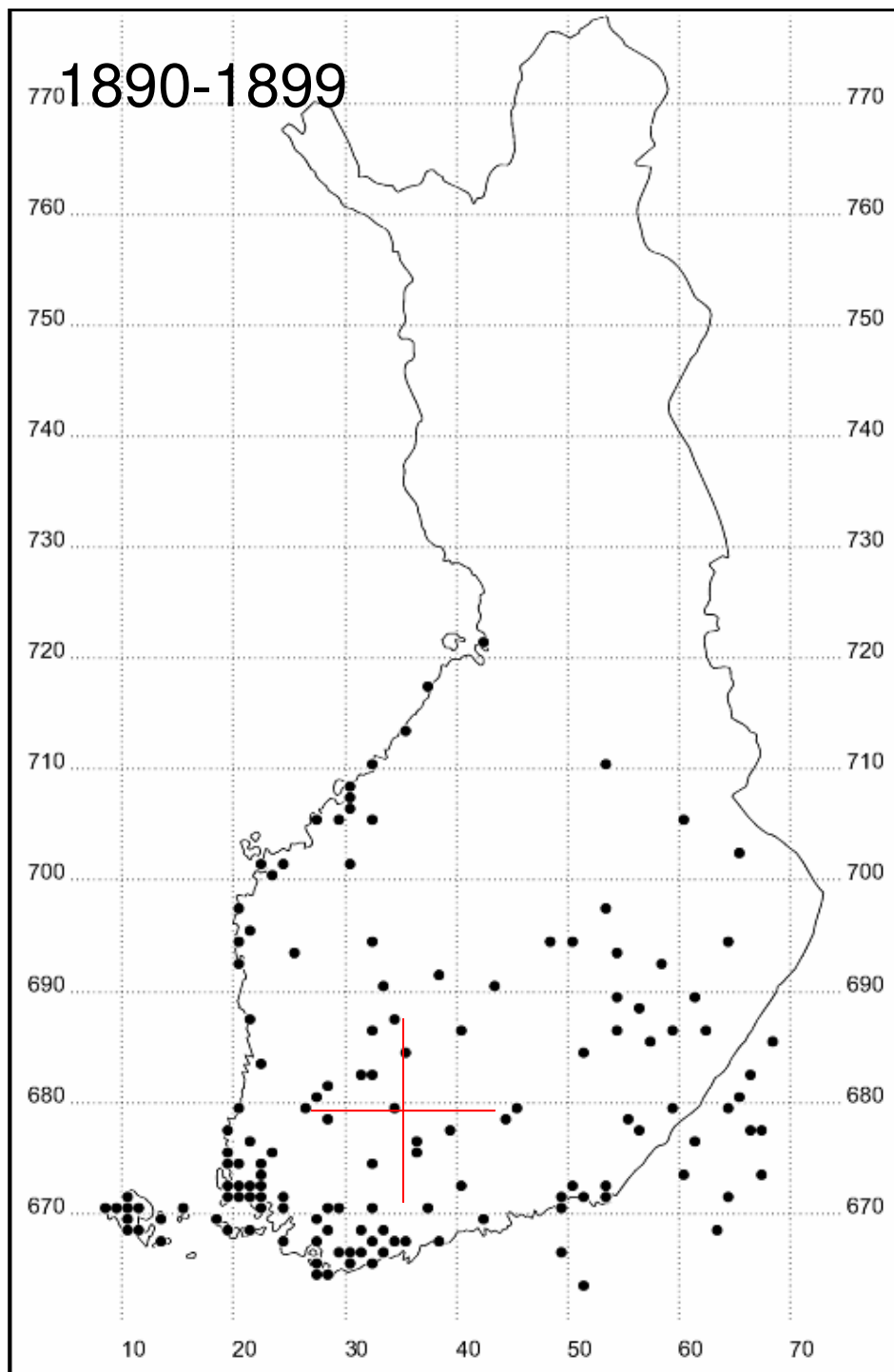

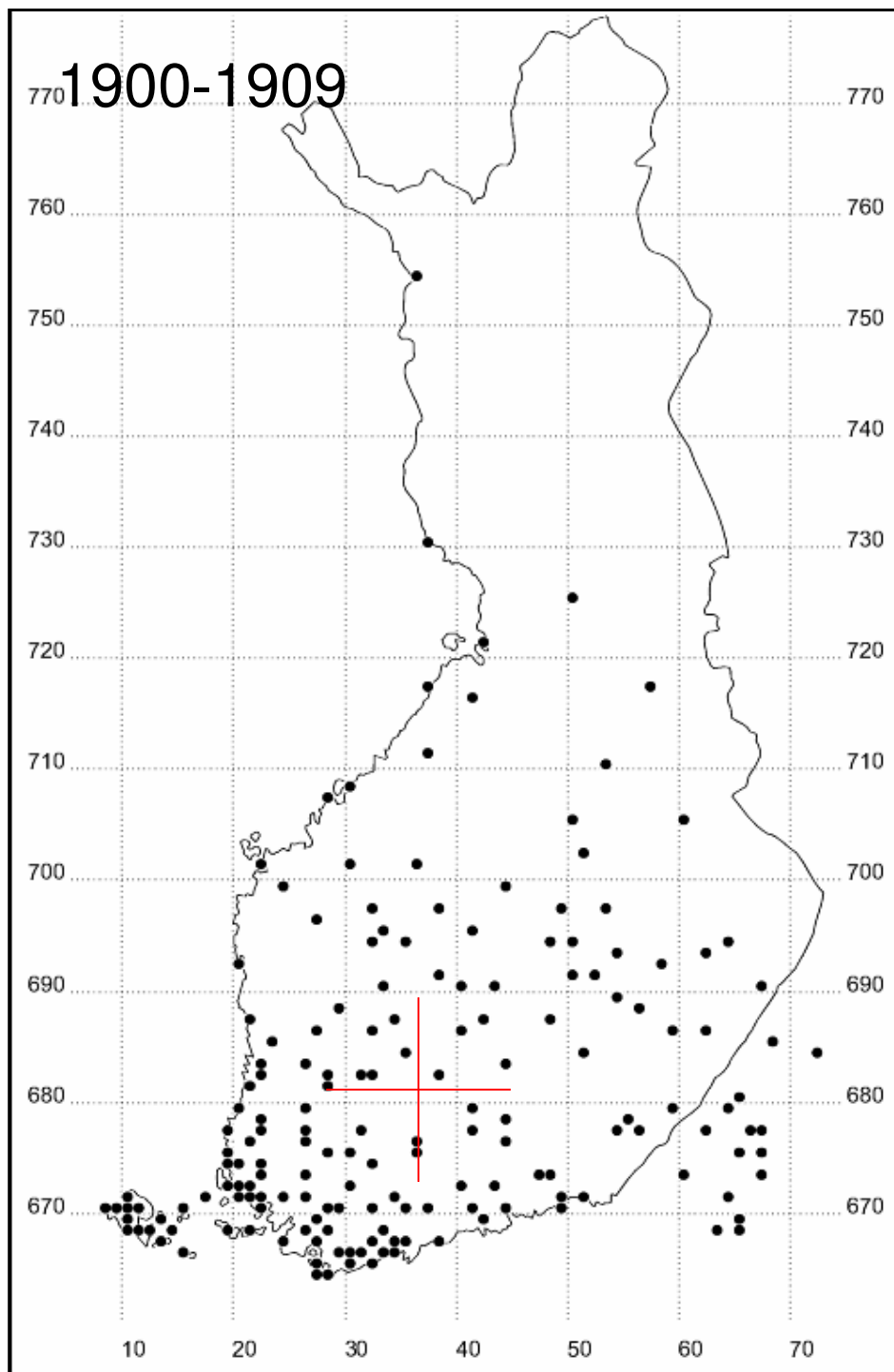

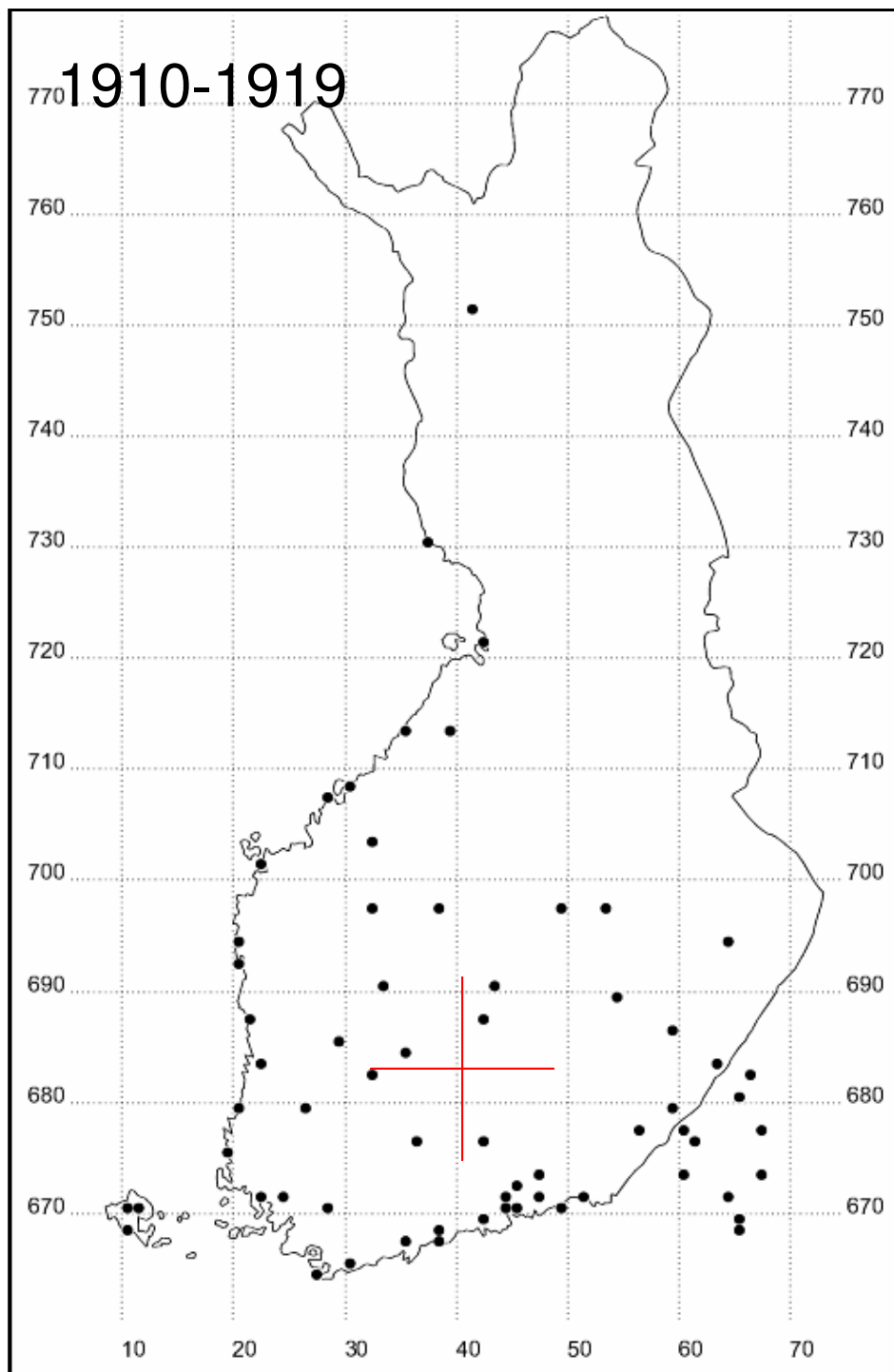

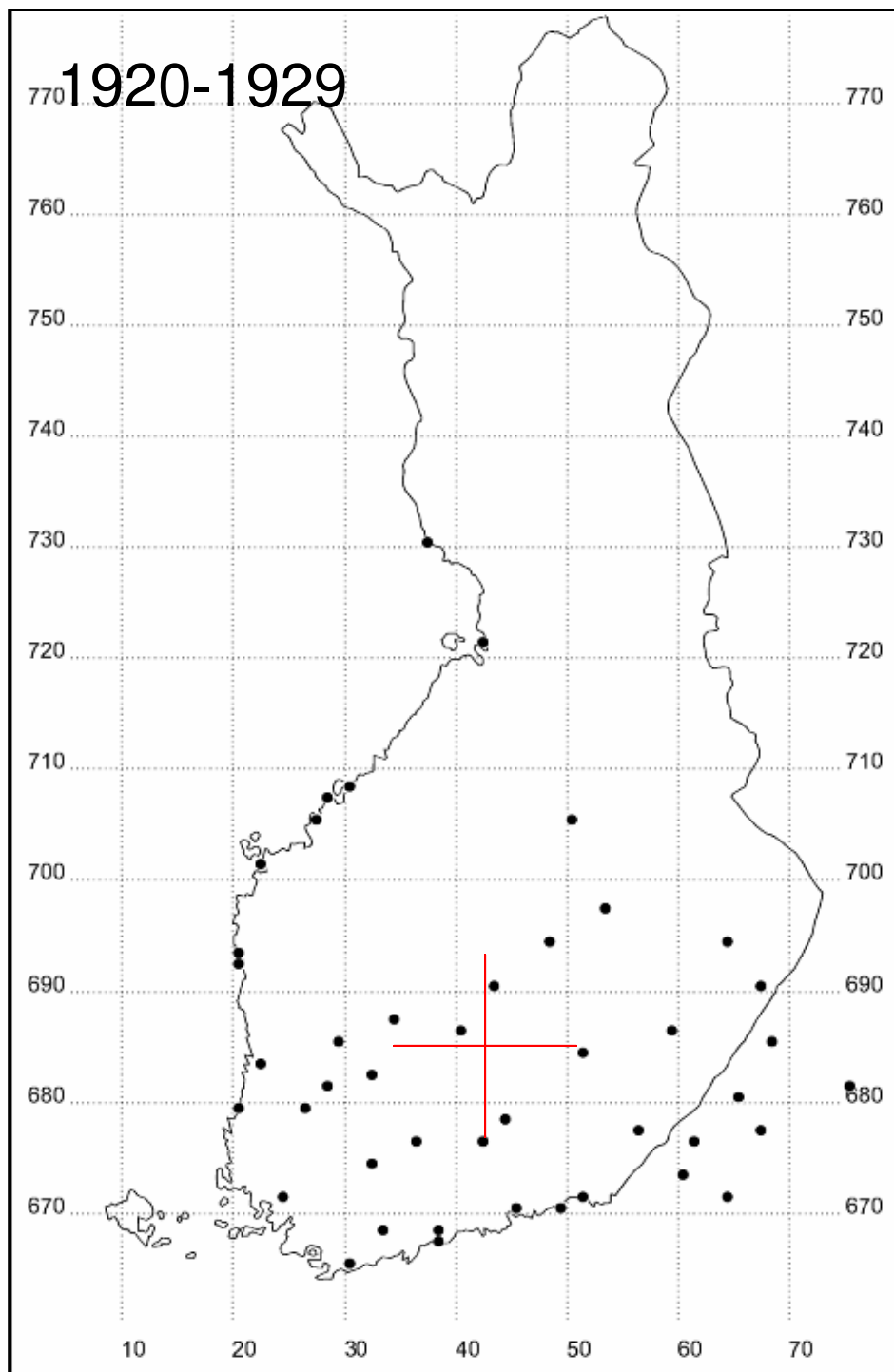

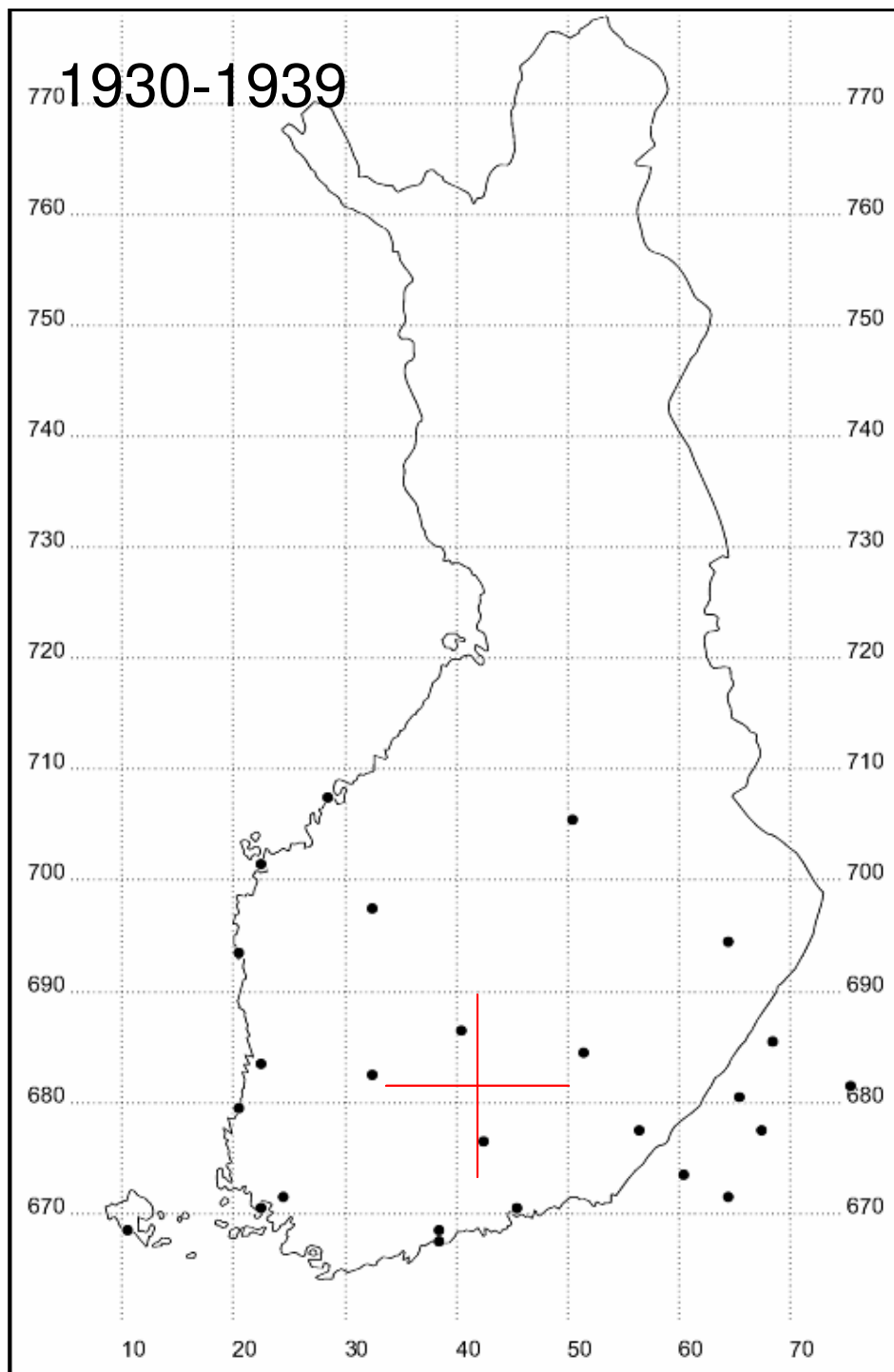

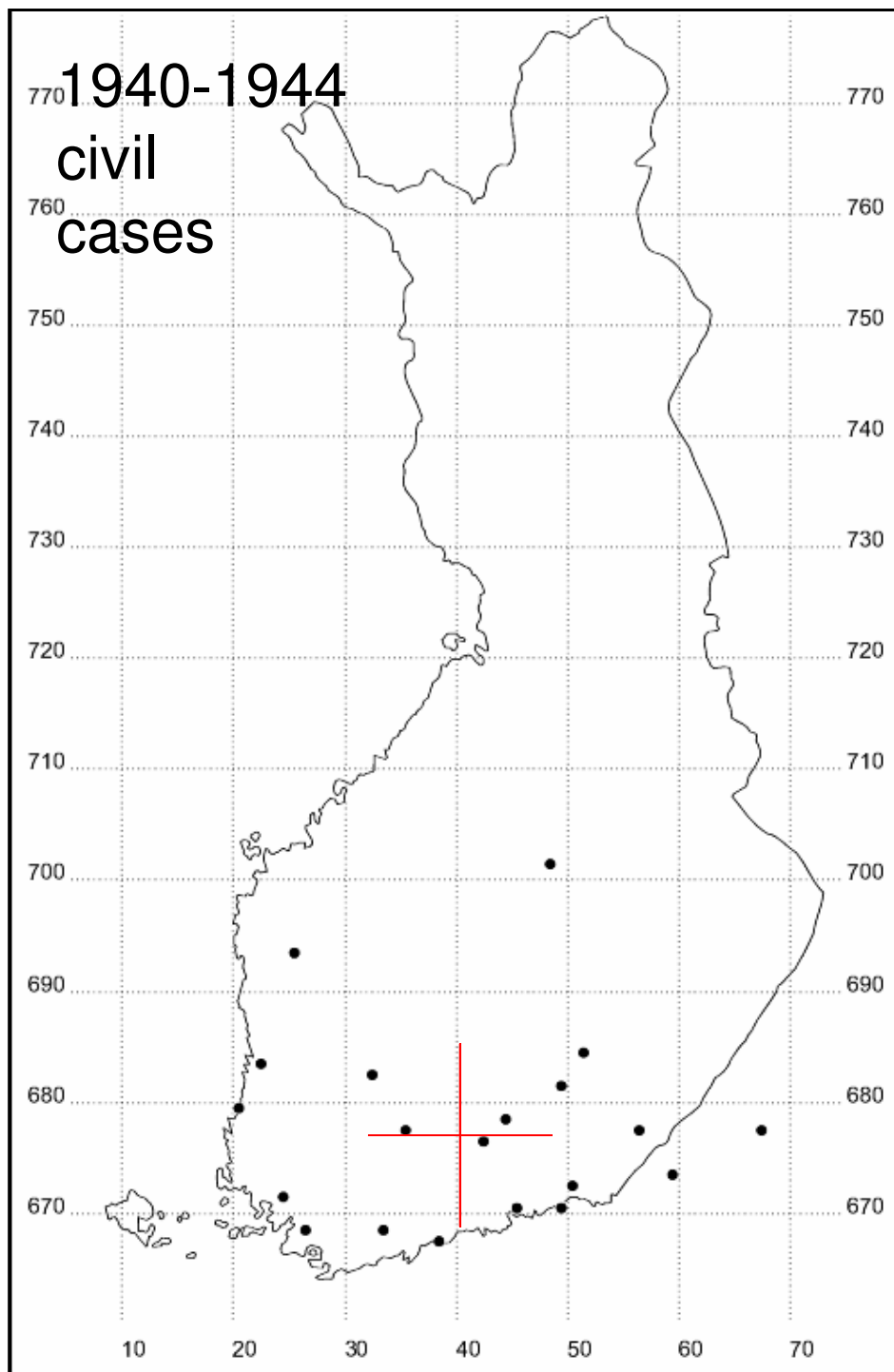

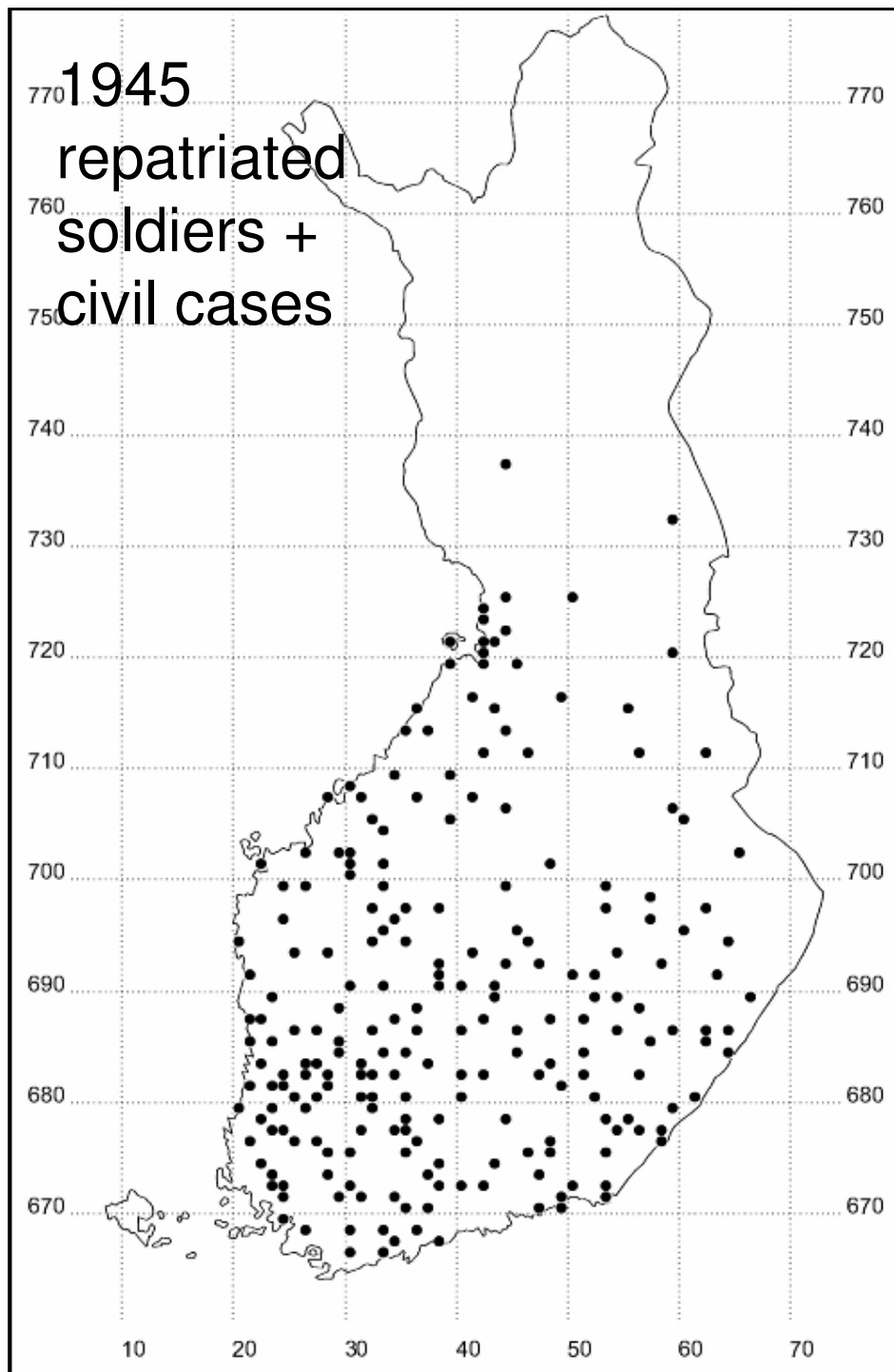

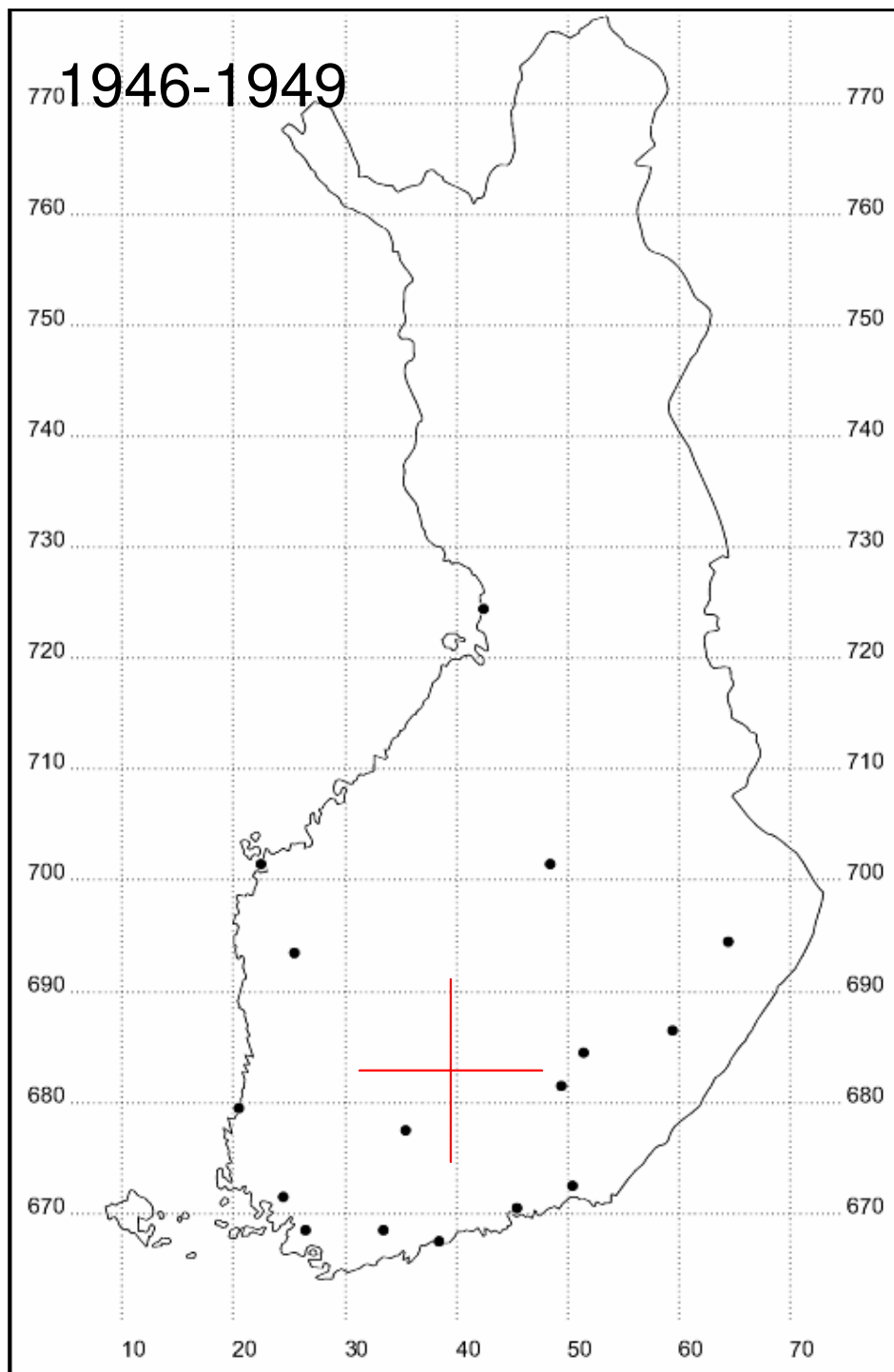

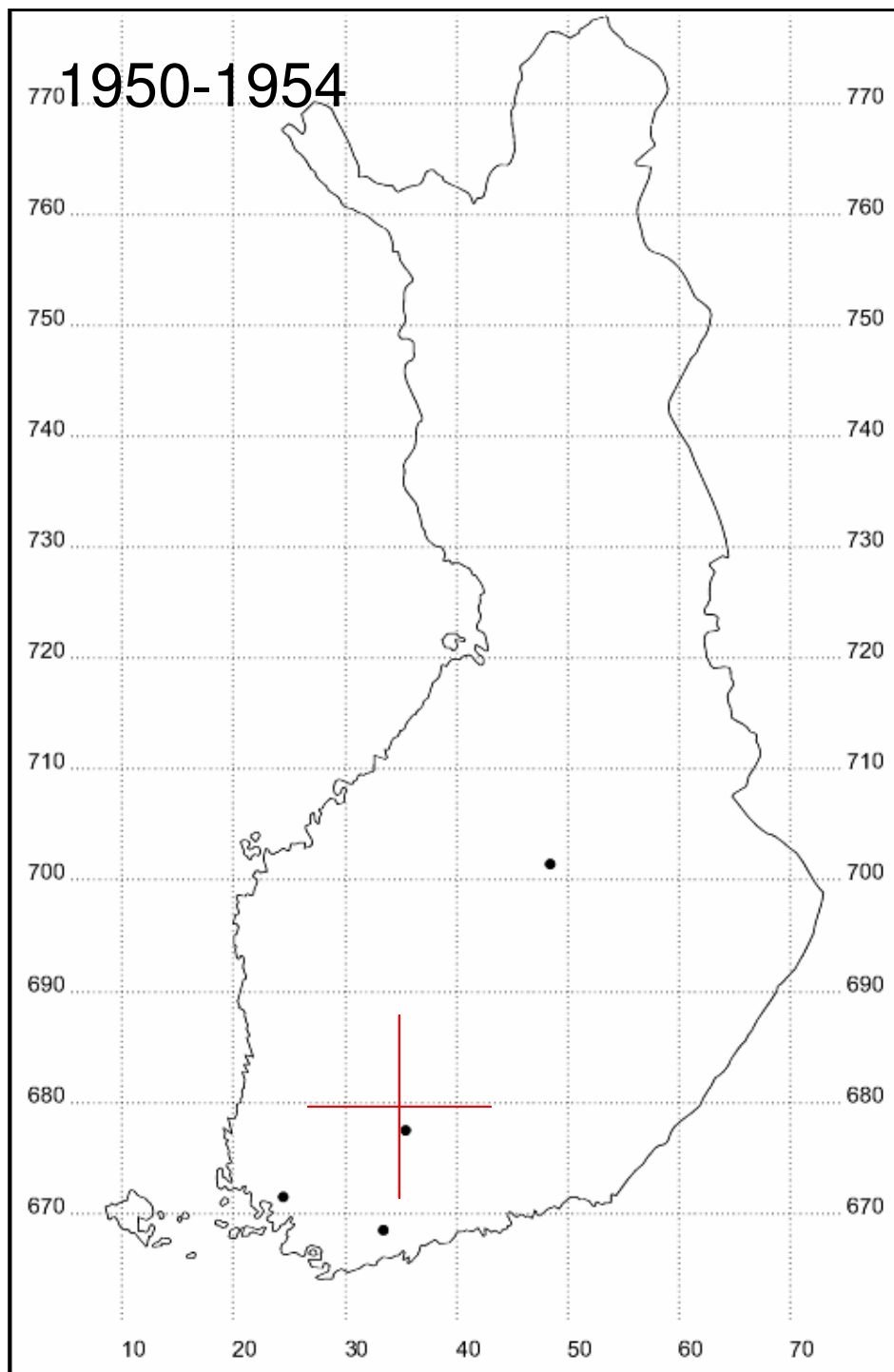

Supplement: Additional File 1 — Decadal distribution of malaria in Finland in 1750–1954. A red cross represents the equilibrium point of all dots on each map. [file 1475-2875-8-94-S1.pdf]
